# Supplementary material for: Can FDG-PET/CT imaging be used to predict decline in quality of life in interstitial lung disease? A prospective study of the relationship between FDG uptake and quality of life in a UK outpatient setting
Source: BMJ Open. 2024 May 30;14(5):e081103. doi: 10.1136/bmjopen-2023-081103 (PMC11141197; doi:10.1136/bmjopen-2023-081103)
Supplement: Supplementary data [file bmjopen-2023-081103supp001.pdf]

Supplemental Materials

Table S1: Mean SGRQ scores for the total study population at each time point

	**Total**	**Symptoms**	**Activity**	**Impact**
**T0**	49.8	54.7	64.1	39.9
**T12**	48.6	51.0	64.7	36.4
**T24**	48.7	50.8	64.2	38.3
**T48**	48.9	52.2	66.2	39.4

Table S2: Correlation between change in PFTs from baseline and baseline FDG-PET parameters

**Correlation between change in FVC from baseline and SUVmax (number of participants)**		
	**Correlation Coefficient (r_s)**	**P value**
**Baseline** (185)	-0.176	0.017
**12 months** (119)	0.010	0.912
**24 months** (73)	0.144	0.225
**48 months** (15)	0.220	0.427
**Correlation between change in TLCO from baseline and SUVmax (number of participants)**		
	**Correlation Coefficient (r_s)**	**P value**
**Baseline** (163)	-0.158	0.043
**12 months** (90)	0.072	0.503
**24 months** (52)	0.030	0.831
**48 months** (10)	0.185	0.604
**Correlation between change in FVC from baseline and TBR (number of participants)**		
	**Correlation Coefficient (r_s)**	**P value**
**Baseline** (185)	-0.003	0.967
**12 months** (119)	-0.101	0.273
**24 months** (73)	0.027	0.818
**48 months** (15)	-0.143	0.611
**Correlation between change in TLCO from baseline and TBR (number of participants)**		
	**Correlation Coefficient (r_s)**	**P value**
**Baseline** (163)	-0.036	0.648
**12 months** (90)	-0.052	0.625
**24 months** (52)	-0.046	0.748
**48 months** (10)	-0.098	0.789

Table S3: Total and subscale SGRQ scores at each time point for the IPF subgroup

	**Total**	**Symptoms**	**Activity**	**Impact**
**T0**	48.8	57.1	63.1	37.8
**T12**	51.1	58.5	66.1	39.3
**T24**	52.8	56.6	67.7	42.8
**T48**	58.8	60.8	72.7	48.7

Table S4: Total and subscale SGRQ scores at each time point for the non-IPF subgroup

	**Total**	**Symptoms**	**Activity**	**Impact**
**T0**	50.8	52.3	65.1	41.9
**T12**	46.4	44.6	63.3	33.8
**T24**	45.5	46.5	61.6	35.3
**T48**	39.6	43.4	59.1	30.1
