# Supplementary material for: Can FDG-PET/CT imaging be used to predict decline in quality of life in interstitial lung disease? A prospective study of the relationship between FDG uptake and quality of life in a UK outpatient setting
Source: BMJ Open. 2024 May 30;14(5):e081103. doi: 10.1136/bmjopen-2023-081103 (PMC11141197; doi:10.1136/bmjopen-2023-081103)
Supplement: Author's manuscript [file bmjopen-2023-081103.draft_revisions.pdf]

BMJ Open

BMJ Open is committed to open peer review. As part of this commitment we make the peer review history of every article we publish publicly available.

When an article is published we post the peer reviewers' comments and the authors' responses online. We also post the versions of the paper that were used during peer review. These are the versions that the peer review comments apply to.

The versions of the paper that follow are the versions that were submitted during the peer review process. They are not the versions of record or the final published versions. They should not be cited or distributed as the published version of this manuscript.

BMJ Open is an open access journal and the full, final, typeset and author-corrected version of record of the manuscript is available on our site with no access controls, subscription charges or pay-per-view fees (http://bmjopen.bmj.com).

If you have any questions on BMJ Open's open peer review process please email info.bmjopen@bmj.com

BMJ Open

Can FDG-PET/CT imaging be used to prognosticate in Interstitial Lung Disease? A prospective study of the relationship between FDG-PET/CT measurements and quality of life.

Journal:	*BMJ Open*
Manuscript ID	bmjopen-2023-081103
Article Type:	Original research
Date Submitted by the Author:	25-Oct-2023

Complete List of Authors:	Jordon, Louise Helen; University of Cambridge, ; Cambridge University Hospitals NHS Foundation Trust,<br>Ganeshan, Balaji; UCL Institute of Nuclear Medicine<br>Nadeem, Iftikhar; Cambridge University Hospitals NHS Foundation Trust<br>Hoy, Luke; UCL Institute of Nuclear Medicine<br>Mahdi, Noor; Cambridge University Hospitals NHS Foundation Trust,<br>Porter, Joanna; UCLH, ILD Centre; UCL, Respiratory Medicine<br>Groves, Ashley; UCL Institute of Nuclear Medicine<br>Win, Thida; Lister Hospital

Keywords:	Interstitial lung disease < THORACIC MEDICINE, NUCLEAR MEDICINE, Quality of Life

SCHOLARONE™
Manuscripts

I, the Submitting Author has the right to grant and does grant on behalf of all authors of the Work (as defined in the below author licence), an exclusive licence and/or a non-exclusive licence for contributions from authors who are: i) UK Crown employees; ii) where BMJ has agreed a CC-BY licence shall apply, and/or iii) in accordance with the terms applicable for US Federal Government officers or employees acting as part of their official duties; on a worldwide, perpetual, irrevocable, royalty-free basis to BMJ Publishing Group Ltd ("BMJ") its licensees and where the relevant Journal is co-owned by BMJ to the co-owners of the Journal, to publish the Work in this journal and any other BMJ products and to exploit all rights, as set out in our licence.

The Submitting Author accepts and understands that any supply made under these terms is made by BMJ to the Submitting Author unless you are acting as an employee on behalf of your employer or a postgraduate student of an affiliated institution which is paying any applicable article publishing charge ("APC") for Open Access articles. Where the Submitting Author wishes to make the Work available on an Open Access basis (and intends to pay the relevant APC), the terms of reuse of such Open Access shall be governed by a Creative Commons licence – details of these licences and which Creative Commons licence will apply to this Work are set out in our licence referred to above.

Other than as permitted in any relevant BMJ Author's Self Archiving Policies, I confirm this Work has not been accepted for publication elsewhere, is not being considered for publication elsewhere and does not duplicate material already published. I confirm all authors consent to publication of this Work and authorise the granting of this licence.

For peer review only

Can FDG-PET/CT imaging be used to prognosticate in Interstitial Lung Disease? A prospective study of the relationship between FDG-PET/CT measurements and quality of life.

Dr Louise Jordon[1,2] *, Dr Balaji Ganeshan[3], Dr Iftikhar Nadeem[4], Luke Hoy[3], Dr Noor Mahdi[4], Prof Joanna C Porter[5,6], Prof Ashley Groves[3], Prof Thida Win[7]

[1]Department of Respiratory Medicine, Cambridge University Hospitals, Cambridge, CB2 0QQ (UK), [2]Jeffrey-Cheah Biomedical Centre, University of Cambridge, Cambridge, CB2 0AW (UK), [3]Institute of Nuclear Medicine, University College London Hospital (UCLH), London, NW1 2PG (UK), [4]Department of Respiratory Medicine Royal Papworth NHS Foundation Trust, Cambridge, CB2 0AY (UK), [5]Department of Respiratory Medicine, University College London, London, WC1E 6JF (UK), [6]ILD Centre, UCLH, London, WC1E 6JF (UK), [7]Department of Respiratory Medicine, Lister Hospital , Stevenage, SG1 4AB (UK).

*Corresponding author: Dr Louise Jordon, University of Cambridge Department of Medicine, Jeffrey Cheah Biomedical Centre, Puddicombe Way, Cambridge, CB2 0QQ. Email: louise.jordon@nhs.net, 07982713087

For peer review only

ABSTRACT

Background

FDG-PET/CT imaging has been used in many inflammatory and infectious conditions to differentiate areas of increased metabolic activity. FDG-PET/CT uptake differs between areas of normal lung parenchyma and interstitial lung disease (ILD).

Objectives

In this study we investigated whether FDG-PET/CT parameters were associated with change in quality of life (QoL) in patients with ILD over 4 years of follow-up.

Methods

Patients underwent PET-CT imaging at diagnosis and were followed up with annual QoL assessment using the St George's Respiratory Questionnaire (SGRQ) until death or 4 years of follow-up. Maximum Standard Uptake Value (SUVmax) and Tissue-to-Background Ratio (TBR) were assessed against SGRQ overall and subscale scores.

Results

193 patients (94 Idiopathic Pulmonary Fibrosis; IPF and 99 non-IPF patients underwent baseline FDG-PET/CT imaging and QoL assessment. Moderate correlation was observed between baseline SUVmax and SGRQ scores in both ILD subgroups. No relationship was observed between baseline SUVmax or TBR and change in SGRQ scores over 4 years of follow-up. In the IPF subgroup, surviving patients reported a decline in QoL at 4 years post diagnosis whereas an improvement in QoL was seen in surviving non-IPF patients.

Conclusions

Weak-to-moderate positive correlation between baseline SUVmax and SGRQ scores was observed in both ILD groups (IPF:r_s=0.191, p=0.041, non-IPF: r_s=0.320, p=0.001). However, baseline SUVmax and TBR were not associated with change in QoL in patients with IPF and non-IPF ILD over 4 years of follow-up. At 4 years post diagnosis, surviving patients with IPF reported declining QoL whereas improvement was seen in non-IPF patients.

Key words:

Interstitial lung disease, idiopathic pulmonary fibrosis, nuclear medicine, quality of life

Strengths and Limitations:

Strengths:

- Large sample of patients with idiopathic pulmonary fibrosis (IPF) and non-IPF ILD.
- 4 years of quality of life follow up data for patients with good questionnaire return rates in surviving patients

Limitations:

- Treatment and specific ILD subtype (aside from IPF) data not collected

For peer review only - http://bmjopen.bmj.com/site/about/guidelines.xhtml

INTRODUCTION

Interstitial Lung Diseases (ILD) are a heterogenous group of pulmonary conditions characterized by varying degrees of fibrosis and inflammation of the lung parenchyma. ILDs are most frequently diagnosed by a combined clinical and radiological assessment [1]. Of these, Idiopathic Pulmonary Fibrosis (IPF) and Non-Specific Interstitial Pneumonia (NSIP) are the most frequently reported, with progressive fibrosis as the dominant histopathological lesion in IPF, and areas of inflammation and, in some cases, fibrosis occurring in NSIP [2].

Despite anti-fibrotic treatments, the prognosis for IPF is poor, with a mean survival of 2.5-5 years from diagnosis [3]. Quality of life (QoL) in IPF is affected by severe breathlessness and activity impairment [4], which demonstrates a close relationship with changes in lung function [5]. Prognosis and symptom burden in NSIP are much better, with an 86-98% 5-year survival for certain subtypes [6].

At diagnosis and during follow-up, it is difficult to predict prognosis for patients with ILD. Decline in Forced Vital Capacity (FVC) is most frequently used to monitor disease progression [7], however baseline FVC is not predictive of change in QoL [5]. Routinely used biomarkers are lacking [8], and so tools to accurately predict QoL deterioration are needed.

Positron Emission Tomography (PET) combined with Computed Tomography (CT) offers a minimally invasive investigation to locate and quantify cellular metabolism. The most frequently used PET tracer is a glucose analogue called fluorodeoxyglucose (FDG). When labelled with the radioisotope 18-Fluorine (^{18}F), it can be used to visualise areas of regional glucose uptake and indicate metabolic activity [9-11]. Over-expression of glucose transporter 1 receptors in malignant cells leads to more FDG uptake, reflecting the increased metabolic activity of cancer cells [11]. More recently, ^{18}F-FDG PET/CT imaging has been used in infectious and inflammatory conditions, where increased FDG uptake is observed due to inflammatory response [12].

In IPF lung tissue, increased glycolysis has been observed in fibroblasts [13], with increased FDG uptake seen in areas of honeycombing [14, 15]. Furthermore, FDG uptake has been shown to be higher in some NSIP patients compared with controls [16], and to correlate with disease activity in sarcoidosis [12], indicating a potential prognostic use for FDG PET in ILD.

Tissue metabolic activity on PET-CT is quantified through Standardised Uptake Values (SUVs) which measure the tracer's radioactivity concentration relative to the dose given and the patient's weight [17]. The SUV is most commonly reported as the maximum SUV within the region of interest (SUVmax) [18]. The tissue-to-background ratio (TBR) is a measure of variation of FDG uptake within the lung, where a value close to 1 indicates relatively uniform uptake [19]. TBR has been used in recent studies as a surrogate marker for inflammation [20, 21], and has been independently associated with a greater risk of mortality in IPF [19].

The primary aim of this study was to investigate any relationship between baseline FDG-PET signal (measured by SUVmax and TBR) and QoL (as a self-reported

For peer review only - http://bmjopen.bmj.com/site/about/guidelines.xhtml

questionnaire) at time of diagnosis and longitudinally over a four year follow up period in patients with ILD, to determine the utility of ^{18}F-FDG PET/CT in ILD prognostication.

For peer review only

For peer review only - http://bmjopen.bmj.com/site/about/guidelines.xhtml

MATERIALS AND METHODS

Study setting and patient selection

This was a prospective, single-centre study from one UK hospital. Patients were recruited from a general respiratory clinic and enrolled for 4 years during the study period from November 2009 to December 2020.

All patients underwent full clinical assessment including multidisciplinary team (MDT) review, baseline pulmonary function tests, and high-resolution CT (HRCT) evaluation. Infection and neoplasia were excluded on clinical and radiological grounds. The diagnosis of IPF was made by MDT review and was based upon HRCT findings of a "usual interstitial pneumonia" (UIP) pattern with no associated cause. If patients did not meet these diagnostic criteria, they were grouped as non-IPF ILD. Written informed consent was obtained for all participants.

Patients had an ^{18}F-FDG PET/CT scan and completed a concurrent baseline QoL questionnaire. They were subsequently followed for 48-months with QoL questionnaires at 12, 24, and 48-month intervals. Patients were excluded if they did not complete a valid QoL questionnaire at baseline, if the SUVmax and TBR could not be calculated or if a diagnosis of IPF or non-IPF ILD could not be reached. A valid questionnaire was one completed in accordance with instructions, with enough questions answered to generate a score.

In total, 234 patients with ILD were recruited and had ^{18}F-FDG PET/CT scans with quantification of SUVmax and TBR. 41 patients were excluded as they did not complete a valid baseline questionnaire, leaving 193 patients in the study.

Study objective

The primary aim of this study was to investigate the relationship between baseline ^{18}F-FDG PET/CT pulmonary uptake (SUVmax and TBR) and QoL measures, assessed using the St George's Respiratory Questionnaire (SGRQ) [22], at the time of the scan and at 12, 24 and 48 months post baseline.

Secondary objectives included looking for correlation between FDG uptake and QoL subscale scores in the total study population and in IPF and non-IPF groups, and observing QoL trajectory over the study period.

QoL data collection

The SGRQ is a disease-specific questionnaire designed to measure the impact on overall health, daily life, and perceived well-being in patients with respiratory disease [22, 23]. It has 50 questions divided into three sections covering: respiratory symptoms, activities that cause or are limited by breathlessness and the psycho-social

For peer review only - http://bmjopen.bmj.com/site/about/guidelines.xhtml

impact of disease. Each question has an empirically derived weight [22], and results in a subsection and overall score out of 100, with higher scores reflecting increased severity. The SGRQ has been well validated in UK and international populations [22, 23].

Baseline SGRQ data was collected when patients attended for their 18F-FDG PET/CT scan (T0), and then at 12, 24 and 48-months later (T12, T24, T48, respectively), by postal questionnaire. QoL trends were calculated by subtracting the baseline QoL score from the score at a subsequent timepoint, thus a positive or negative score indicated a deterioration or improvement respectively.

FDG PET-CT imaging data collection

The 18F-FDG PET/CT was performed as previously described [19].

Patients fasted for 6 hours. Images were acquired 1 hour after an injection of ^{18}F-FDG (200MBq) on a dedicated combined PET/64-detector CT scanner (GE Healthcare). Three sequential scans of the thorax were performed while the patient remained supine on the table with their arms above their head. First, an attenuation-correction CT (CTAC) was performed using 64 3.75 mm detectors, a pitch of 1.5, and a 5-mm collimation (140kVp and 80mA in 0.8s).

Patient position was maintained while a whole-body ^{18}F-FDG PET scan was acquired, covering an area identical to the CTAC. All images were acquired in 2-dimensional mode (8min/bed position). Transaxial emission images of 3.27mm thickness (pixel size 3.9mm) were reconstructed using ordered-subsets expectation maximization with 2 iterations and 28 subsets. The axial field of view was 148.75mm, resulting in 47 slices per bed position. Next, a deep inspiratory HRCT was performed whilst maintaining patient position, using 64 1.25 mm detectors, pitch of 0.53, and 1.25-mm collimation (120kVp and 100mAs).

Image Analysis

PET/CT images were reviewed by 2 combined radiologist/nuclear medicine physicians in consensus, one of whom had a specialist interest in cardiothoracic PET/CT. PET/CT images were loaded onto an Xeleris workstation (GE Healthcare Technology). Both attenuation and non–attenuation-corrected images were reviewed to ensure that areas of higher-density lung were not inducing attenuation-correction artifacts. The area of most intense pulmonary FDG uptake was identified and SUVmax measured. The HRCT parenchymal pattern in the region was also assessed. In addition, SUV_{min} was used to determine the background lung uptake, and in turn calculate TBR.

Statistical Analysis:

Statistical analyses were performed using SPSS v26 (IBM) and Prism v8 (GraphPad). A two-sided p-value of less than 0.05 indicated a significant relationship. The

For peer review only - http://bmjopen.bmj.com/site/about/guidelines.xhtml

relationship between FDG-PET/CT uptake and QoL was assessed using non-parametric Spearman's rank correlation.

Patient and public involvement statement:

Whilst patients were not involved directly in the design of this study, the research question and outcome measures were focused upon QoL rather than lung function or other physical parameters in line with patients' greatest priorities.

For peer review only

For peer review only - http://bmjopen.bmj.com/site/about/guidelines.xhtml

RESULTS

Total population

Baseline characteristics

In total, 193 patients met the inclusion criteria (120 males, 59 females, 14 missing; average age ± SD: 66.6+/-9 years). **Table 1** shows baseline characteristics

Table 1: Baseline characteristics of the study population

	Data
**Males (%)**	120 (67.0)
**Mean Age (SD)**	66.6 (9)
**Diagnosis: IPF (%)**	94 (48.7)
**NSIP (%)**	78 (40.4)
**Non-NSIP (%)**	21 (10.9)
**Mean baseline FEV1 (SD; range)**	74.9 (18.7; 17-122)
**Mean baseline FVC (SD; range)**	75.8 (17.6; 20-129)
**Mean baseline TLCO (SD; range)**	46.7 (15.5; 11-98)

The mean values for the baseline PET uptake (range in brackets) for the population were: SUVmax 3.25 (1.4-7.4), SUVmin 0.55 (0.16-1.3), and TBR 6.28 (2.4-15.3).

QoL assessment

QoL data was available for all 193 patients at the time of recruitment (T0), 99 patients at T12 (51.3%), 91 patients at T24 (47.2%) and 45 patients at T48 (23.3%). During follow-up of the 193 patients, 21 died by 12 months (10.9%), 42 by 24 months (21.8%) and 120 by 48 months (62.2%).

Table S1 (supplemental materials) demonstrates the mean QoL scores for the population who returned the SGRQ at the study time points, displayed as a percentage of the total possible score.

There was no significant difference in total SGRQ scores at T0 and T12 (t=0.44, p=0.66), T24 (t=0.36, p=0.72) or T48 (t=0.2, p=0.84).

Relationship between SGRQ and FDG-PET

Example PET-CT images with their corresponding SGRQ scores are shown in **Figure 1A-D.**

At baseline, there was weak positive correlation between SUVmax and total SGRQ score (r_s=0.219, p=0.002) and the SGRQ Symptom, Activity, and Impact subscales (r_s=0.211, 0.178 and 0.209 respectively, p<0.003 for all). There was no significant relationship between TBR and total (r_s=-0.015, p=0.841) and subscale SGRQ scores.

When change in QoL over time was assessed, there was no significant correlation between change in total SGRQ score and baseline SUVmax at 12 months (r_s = -0.084, p=0.403), 24 months (r_s=0.184, p-0.084) and 48 months (rs=0.084, p=0.56).

For peer review only - http://bmjopen.bmj.com/site/about/guidelines.xhtml

Additionally, there was no relationship between change in SGRQ subscale scores at each time point and SUVmax.

We also observed no significant correlation between change in total SGRQ score and baseline TBR at T12 (r_s= -0.065, p=0.519), T24 (r_s= -0.014, p=0.893) and T48 (r_s= -0.113, p=0.435), and no relationship between change in SGRQ subscale scores at each time point and TBR.

IPF- ILD subset

Baseline demographics

There were 94 IPF patients (74 males, 14 females, 6 missing; average-age ± SD: 69.5+/-8.9 years) in this subgroup.

The mean (range) of baseline PET metrics were: SUVmax 3.4 (1.8-6.0) and TBR 5.4 (3.1-16.6).

QoL assessment

QoL data was available in all 94 patients at T0. At 12 months 47 patients (50%) returned the SGRQ, 40 (42.5%) at 24 months and 22 (23.4%) at 48 months. During the follow-up period for the 94 patients, 13 died by 12 months (13.8%), 24 by 24 months (25.5%) and 74 by 48 months (78.7%).

Table S2 (supplemental materials) shows the total and subscale SGRQ scores at each time point for the IPF subgroup

When change in QoL was assessed over time, there was no significant difference in total SGRQ at T0 and T12 (t=0.74, p=0.46) or T0 and T24 (t=1.23, p=0.22) but a significant increase in total SGRQ, indicating deteriorating QoL, was seen at 48 months (t=-3.1. p=0.002).

Relationship between SGRQ scores and FDG-PET

At baseline, there was weak positive correlation between total SGRQ score and SUVmax (r_s=0.187, p=0.047; **Figure 2**). There was also weak positive correlation between the Activity SGRQ subscale and SUVmax (r_s= 0191, p=0.039). There was no significant relationship between TBR and total (r_s=0.061, p=0.524) and subscale SGRQ scores.

When change in QoL over time was assessed against pulmonary FDG-PET uptake, there was no significant correlation between change in total SGRQ score and baseline SUVmax at T12 (r_s=0.065, p =0.653), T24 (r_s=0.017, p=0.913) and T48 (r_s=0.084, p=0.684; Figure 1). Additionally, there was no relationship between change in SGRQ subscale scores at each time point and SUVmax.

We also observed no significant correlation between change in total SGRQ score and baseline TBR at T12 (r_s=0.035, p=0.807), T24 (r_s= -0.122, p=0.419) and T48 (r_s=0.006,

For peer review only - http://bmjopen.bmj.com/site/about/guidelines.xhtml

p=0.978, and no relationship between change in SGRQ subscale scores at each time point and TBR.

Non-IPF subset

Baseline demographics

There were 99 Non-IPF ILD patients (46 males, 45 females, 8 missing; mean age ±SD: 64.6+/-10.1 years). The mean (range) of baseline PET uptake metrics were: SUVmax 3.1 (1.4-6.4) and TBR 6.8 (2.9-13.6).

QoL assessment

QoL data was available in all 99 patients at the time of recruitment (T0). 52 patients (52.5%) returned the QoL questionnaire at 12 months, 51 (54.3%) at 24 months and 23 (23.2%) at 48 months. Of the 99 patients, 8 died by 12 months (8.1%), 18 by 24 months (18.1%) and 46 by 48 months (46.5%) of follow up.

Table S3 (supplemental materials) shows the total and subscale SGRQ scores at each time point for the non-IPF subgroup

When change in QoL was assessed over time, there was no significant difference in total SGRQ at T0 and T12 (t=1.14, p=0.26) or T0 and T24 (t=1.23, p=0.22) but a significant decrease in total SGRQ, indicating improved QOL, was seen at 48 months (t=2.04. p=0.05).

In comparison with total SGRQ scores from the IPF subgroup, there was no significant difference at T0 (t=0.614, p=0.54), T12 (t=1.09, p=0.28) or T24 (t=1.43, p=0.16). Scores in the non-IPF group were significantly lower than those of the IPF group at T48 (t=-3.15, p=0.003). Comparison of overall SGRQ scores between the IPF and non-IPF subgroups is shown in **Figure 3.**

Relationship between SGRQ scores and FDG-PET

At baseline there was moderately positive correlation with total SGRQ score and SUVmax (r_s=0.320, p=0.001; **Figure 4**). There was also positive correlation between the baseline Symptoms, Activity and Impact subscale scores and baseline SUVmax (r_s= 0.287, 0.242 and 0.314, p<0.008 respectively). There was no significant relationship between TBR and total (r_s=-0.085, p=0.379) and subscale SGRQ scores.

When change in QoL over time was assessed against pulmonary FDG PET uptake, there was no significant correlation between change in total SGRQ score and baseline SUVmax at T12 (r_s= -0.126, p=0.347), T24 (r_s=0.188, p=0.188) and T48 (r_s= -0.121, p=0.539; Figure 3). Additionally, there was no relationship between change in SGRQ subscale scores at each time point and baseline SUVmax.

We also observed no significant correlation between change in total SGRQ score and baseline TBR at T12 (r_s= -0.051, p=0.702), T24 (r_s=0.075, p=0.603) and T48 (r_s=0.083,

For peer review only - http://bmjopen.bmj.com/site/about/guidelines.xhtml

p=0.676, and no relationship between change in SGRQ subscale scores at each time point and TBR.

For peer review only

For peer review only - http://bmjopen.bmj.com/site/about/guidelines.xhtml

DISCUSSION

The aim of this study was to investigate the relationship between ^{18}FDG-PET/CT metrics and QoL in patients with IPF and non-IPF ILD to determine if ^{18}FDG-PET/CT could be used in ILD prognostication. To our knowledge, this study is the largest to date investigating this relationship.

Relationship between FDG-PET/CT and QoL

Our study showed that there was weak association between baseline SUVmax and QoL measured by the SGRQ. This may indicate that the metabolic activity of ILD may relate to a patient's symptoms and QoL. However, in both IPF and non-IPF subgroups, we observed no significant relationship between baseline SUVmax or TBR and change in QoL over the 48-month follow-up period. As such, these baseline PET measurements do not appear to be associated with patient-reported change in QoL for either ILD subgroup.

Work from our group has previously shown higher TBR to be predictive of survival in patients with IPF [24]. More recently we have also shown TBR to correlate with biomarkers of new vessel angiogenesis, which itself is predictive of poorer survival [25]. In 27 patients with IPF, SUVmax was shown to be predictive of FVC, TLCO and physiological decline at 12 months [15]. A larger study of 89 patients reported baseline SUVmax to be predictive of TLCO decline at 6 months, but no relationship was observed with survival outcomes [26].

However, existing literature on FDG-PET/CT as a prognostic tool in IPF is mixed. Groves *et al* [14] observed a relationship between baseline SUVmax and baseline global health and lung function in 18 patients with IPF, but these measures were not predictive of decline at 6 months. This concurs with our findings of correlation between baseline SUVmax and QoL measures in IPF patients. When evaluating the role of 18F-FDG PET/CT in predicting treatment response in IPF, Bondue *et al* found SUVmax to be predictive of pirfenidone response in a mouse bleomycin model, but not in 25 patients with IPF, and nor was it predictive of disease progression during the 3-month study period in the patient population [27].

Small previous studies have shown FDG uptake distribution in NSIP, but not SUVmax to be predictive of response to treatment [16]. In patients with advanced systemic sclerosis associated ILD, ^{18}F-FDG PET/CT was unable to differentiate between areas of active inflammation (ground glass opacities on HRCT) and areas of fibrosis, but could differentiate between normal lung parenchyma and ILD changes.

To our knowledge, this is the first study evaluating the relationship between patient reported QoL and SUVmax and TBR. Whilst some studies have shown a relationship between these parameters and lung function or survival, we may not have seen a relationship between them and QoL trajectory because QoL has many more contributing factors. Furthermore, the drop out in questionnaire returns over our study period is due to patient death, and hence only patients with a milder disease trajectory were evaluated at our 48-month time point. Additionally, whilst baseline FEV1 and FVC measures of our patients were similar to those in other studies [15], our patients were enrolled from diagnosis whereas other studies enrolled subjects at later disease stages. Finally, studies evaluating PET-CT as a prognostic tool have focused on

For peer review only - http://bmjopen.bmj.com/site/about/guidelines.xhtml

different PET parameters such as FDG uptake pattern and SUVmean, and hence using these may have led to an observed relationship with QoL.

Combining this study with prior work [5, 14, 24], there is presently insufficient evidence to support using baseline FVC [5] or SUVmax/TBR to predict symptomatic decline in ILD [14], but these can be used to predict survival outcomes in IPF-ILD [24].

QoL trajectory in patients with IPF and non-IPF ILD

The trajectory of patients' QoL over the follow-up period was evaluated as one of our secondary outcomes. Better QoL was observed in the non-IPF subgroup compared to the IPF group at all points from 12-48 months follow-up In the IPF subgroup, QoL deterioration was observed at the 48 month time point, but not prior to this. Notably, 78% of the initial IPF cohort had died at 48 months. For the non-IPF group, there was no difference in QoL measures at 12 and 24 months compared with baseline, but a slight improvement seen at 48 months, by which point 46% of the cohort had died. Thus, patients with IPF who survived to 48 months post diagnosis noted a decline in their QoL at this stage, whereas surviving non-IPF ILD patients began to improve at this stage. This enhances our knowledge of known prognostic indicators for different types of ILD. Improving QoL over the longer term in non-IPF patients is an encouraging finding. The non-IPF ILD subgroup represent a clinically heterogenous group. The observation of improvement in QoL in surviving patients is likely due to effective treatment options for certain NSIP subtypes.

Notably, surviving IPF patients did not note a decline in their QoL until 48 months post diagnosis. This may reflect a milder disease phenotype in the surviving patients, or signal an abrupt symptomatic decline in patients with IPF in the couple of years before they die. A large registry study of 277 IPF patients observed SGRQ-measured QoL was static for 12 months and then declined in patients a mean of three years post IPF diagnosis [28]. In particular, QoL has been shown to be very low in the two years prior to death in IPF patients [29]. Our study supports this existing work, and reinforces the need for palliative care input alongside other treatments for IPF patients at this stage of disease.

Limitations and future work

This study benefits from recruiting a large number of patients with 4 years of QoL data and a good questionnaire return rate in surviving patients. Nonetheless, there are some limitations. We did not collect treatment data and hence cannot account for their potential impact. Furthermore, we cannot draw conclusions on specific ILDs and the effect of ^{18}F-FDG PET/CT as a prognostic marker there.

Future work could focus upon PET/CT measures and QoL in specific ILD subgroups, particularly those with an inflammatory phenotype, and could explore the role of PET-CT in treatment response prediction.

For peer review only - http://bmjopen.bmj.com/site/about/guidelines.xhtml

CONCLUSIONS

^{18}F-FDG PET/CT measure SUVmax demonstrated weak-to-moderate correlation with QoL at baseline, but baseline SUVmax and TBR were not associated with change in QoL in patients with IPF and non-IPF ILD. At present, there is insufficient evidence to support the role of ^{18}F-FDG-PET/CT in QoL prediction in ILD. Further prospective studies of ^{18}F-FDG-PET/CT involving treatment data, QoL and other imaging parameters may be beneficial to identify non-invasive disease markers.

At 4 years post IPF diagnosis, surviving patients reported declining QoL, whereas surviving non-IPF ILD patients reported an improvement in their QoL, potentially reflecting the beneficial effect of treatment.

For peer review only

FIGURE CAPTIONS

Figure 1: Example PET-CT images from study patients. Images show the helical acquisition CT scan, attenuation-corrected F18 emission and the axially fused PET-CT (L→R). Boxed area on the scans indicates region of SUVmax. **A:** SUVmax 4.1, SGRQ 72.99, **B:** SUVmax 3.9, SGRQ 58.83, **C:** SUVmax 2.1, SGRQ 7.85, **D:** SUVmax 2.68, SGRQ 15.66.

Figure 2: Relationship between baseline SUVmax and SGRQ at (A) baseline and (B) 48 months follow up in the IPF subgroup

Figure 3: SGRQ by ILD group over 48 month follow up period.

Figure 4: Relationship between baseline SUVmax and SGRQ at (A) baseline and (B) 48 months follow up in the non-IPF subgroup

For peer review only

For peer review only - http://bmjopen.bmj.com/site/about/guidelines.xhtml

ACKNOWLEDGEMENTS
This work was presented at the 2022 European Respiratory Society International Congress in the ILD poster session. *Eur Respir J* 2022; 60: Suppl. 66, 2473. https://doi.org/10.1183/13993003.congress-2022.2473

CONFLICT OF INTEREST STATEMENT
The authors report no conflicts of interest to declare.

FUNDING SOURCES
This study was undertaken at UCLH/UCL who received a proportion of funding from the Department of Health's NIHR Biomedical Research Centre's funding scheme.

STATEMENT OF ETHICS
This study was approved by the Joint UCL/UCLH Committees on the Ethics of Human Research (Committee A)

REC reference number 06/Q0505/22

DATA AVAILABILITY
Data from this study are available upon reasonable request. This comprises deidentified patient demographic and quality of life data, and calculated PET parameters. Images from which PET parameters were calculated may also be made available upon reasonable request.

For peer review only

REFERENCES

[1] K.M. Antoniou, G.A. Margaritopoulos, S. Tomassetti, F. Bonella, U. Costabel, V. Poletti, Interstitial lung disease, Eur Respir Rev 23(131) (2014) 40-54.

[2] R. du Bois, T.E. King, Challenges in pulmonary fibrosis x 5: the NSIP/UIP debate, Thorax 62(11) (2007) 1008-12.

[3] H. Fujimoto, T. Kobayashi, A. Azuma, Idiopathic Pulmonary Fibrosis: Treatment and Prognosis, Clin Med Insights Circ Respir Pulm Med 9(Suppl 1) (2015) 179-185.

[4] J. De Vries, B.L. Kessels, M. Drent, Quality of life of idiopathic pulmonary fibrosis patients, Eur Respir J 17(5) (2001) 954-61.

[5] M. Kreuter, J. Swigris, D. Pittrow, S. Geier, J. Klotsche, A. Prasse *et al.* The clinical course of idiopathic pulmonary fibrosis and its association to quality of life over time: longitudinal data from the INSIGHTS-IPF registry, Respir Res 20(1) (2019) 59.

[6] W.D. Travis, K. Matsui, J. Moss, V.J. Ferrans, Idiopathic nonspecific interstitial pneumonia: prognostic significance of cellular and fibrosing patterns: survival comparison with usual interstitial pneumonia and desquamative interstitial pneumonia, Am J Surg Pathol 24(1) (2000) 19-33.

[7] R.M. du Bois, D. Weycker, C. Albera, W.Z. Bradford, U. Costabel, A. Kartashov, *et al.* Forced vital capacity in patients with idiopathic pulmonary fibrosis: test properties and minimal clinically important difference, Am J Respir Crit Care Med 184(12) (2011) 1382-9.

[8] S. Alqalyoobi, A. Adegunsoye, A. Linderholm, C. Hrusch, C. Cutting, S.F. Ma, *et al.* Circulating Plasma Biomarkers of Progressive Interstitial Lung Disease, Am J Respir Crit Care Med 201(2) (2020) 250-253.

[9] G.I. Bell, C.F. Burant, J. Takeda, G.W. Gould, Structure and function of mammalian facilitative sugar transporters, J Biol Chem 268(26) (1993) 19161-4.

[10] F.J. Gallagher BM , Gutterson NI , et al, Metabolic trapping as a principle of radiopharmaceutical design: some factors responsible for the biodistribution of [18 F] 2-deoxy-2-fluoro- d -glucose . Journal of Nuclear Medicine 19 (1978) 1154-1161.

For peer review only - http://bmjopen.bmj.com/site/about/guidelines.xhtml

[11] E.K. Pauwels, M.J. Ribeiro, J.H. Stoot, V.R. McCready, M. Bourguignon, B. Mazière, FDG accumulation and tumor biology, Nucl Med Biol 25(4) (1998) 317-22.

[12] S. Capitanio, A.J. Nordin, A.R. Noraini, C. Rossetti, PET/CT in nononcological lung diseases: current applications and future perspectives, Eur Respir Rev 25(141) (2016) 247-58.

[13] N. Xie, Z. Tan, S. Banerjee, H. Cui, J. Ge, R.M. Liu,*et al.* Glycolytic Reprogramming in Myofibroblast Differentiation and Lung Fibrosis, Am J Respir Crit Care Med 192(12) (2015) 1462-74.

[14] A.M. Groves, T. Win, N.J. Screaton, M. Berovic, R. Endozo, H. Booth *et al.* Idiopathic pulmonary fibrosis and diffuse parenchymal lung disease: implications from initial experience with 18F-FDG PET/CT, J Nucl Med 50(4) (2009) 538-45.

[15] A. Justet, A. Laurent-Bellue, G. Thabut, A. Dieudonné, M.P. Debray, R. Borie,*et al.* [18 F]FDG PET/CT predicts progression-free survival in patients with idiopathic pulmonary fibrosis, Respir Res 18(1) (2017) 74.

[16] V. Jacquelin, A. Mekinian, P.Y. Brillet, H. Nunes, O. Fain, D. Valeyre, M. Soussan, FDG-PET/CT in the prediction of pulmonary function improvement in nonspecific interstitial pneumonia. A Pilot Study, Eur J Radiol 85(12) (2016) 2200-2205.

[17] P.E. Kinahan, J.W. Fletcher, Positron emission tomography-computed tomography standardized uptake values in clinical practice and assessing response to therapy, Semin Ultrasound CT MR 31(6) (2010) 496-505.

[18] M.C. Adams, T.G. Turkington, J.M. Wilson, T.Z. Wong, A systematic review of the factors affecting accuracy of SUV measurements, AJR Am J Roentgenol 195(2) (2010) 310-20.

[19] T. Win, N.J. Screaton, J.C. Porter, B. Ganeshan, T.M. Maher, F. *Fraioliet al.* Pulmonary 18 F-FDG uptake helps refine current risk stratification in idiopathic pulmonary fibrosis (IPF), Eur J Nucl Med Mol Imaging 45(5) (2018) 806-815.

[20] A.L. Figueroa, S.S. Subramanian, R.C. Cury, Q.A. Truong, J.A. Gardecki, G.J. Tearney *et al.* Distribution of inflammation within carotid atherosclerotic plaques with high-risk morphological features: a comparison between positron emission tomography activity, plaque morphology, and histopathology, Circ Cardiovasc Imaging 5(1) (2012) 69-77.

For peer review only - http://bmjopen.bmj.com/site/about/guidelines.xhtml

[21] A. Honda, N. Tahara, Y. Nitta, A. Tahara, S. Igata, M. Bekki. *et al.* Vascular Inflammation Evaluated by [18F]-Fluorodeoxyglucose-Positron Emission Tomography/Computed Tomography Is Associated With Endothelial Dysfunction, Arterioscler Thromb Vasc Biol 36(9) (2016) 1980-8.

[22] P.W. Jones, F.H. Quirk, C.M. Baveystock, The St George's Respiratory Questionnaire, Respir Med 85 Suppl B (1991) 25-31; discussion 33-7.

[23] M. Ferrer, C. Villasante, J. Alonso, V. Sobradillo, R. Gabriel, G. Vilagut, *et al.* Interpretation of quality of life scores from the St George's Respiratory Questionnaire, Eur Respir J 19(3) (2002) 405-13.

[24] T. Win, N.J. Screaton, J.C. Porter, B. Ganeshan, T.M. Maher, F. Fraioli, *et al.* Pulmonary 18F-FDG uptake helps refine current risk stratification in idiopathic pulmonary fibrosis (IPF), European Journal of Nuclear Medicine and Molecular Imaging 45(5) (2018) 808-818.

[25] J. Porter, B. Ganeshan, T. Win, F. Fraioli, S. Khan, M. Rodriguez-Justo, *et al.* [18 F]FDG PET signal correlates with markers of neo-angiogenesis in patients with fibrotic interstitial lung disease who underwent lung biopsy: Implication for the use of PET/CT in diffuse lung diseases, Preprint: Research Square (2022).

[26] Hee-Young Yoon, Suk Hyun Lee, Sejin Ha , Jin-Sook Ryu , J.W. Song, The Value of 18 F-FDG PET/CT in Evaluating Disease Severity and Prognosis in Idiopathic Pulmonary Fibrosis Patients, 2021, p. 257.

[27] B. Bondue, A. Castiaux, G. Van Simaeys, C. Mathey, F. Sherer, D. Egrise *et al.* Absence of early metabolic response assessed by 18F-FDG PET/CT after initiation of antifibrotic drugs in IPF patients, Respir Res 20(1) (2019) 10.

[28] W.A. Wuyts, C. Dahlqvist, H. Slabbynck, M. Schlesser, N. Gusbin *et al.* Quality of Life and Healthcare Resource Use in a Real-world Patient Population with Idiopathic Pulmonary Fibrosis: The PROOF Registry, Pulm Ther 8(2) (2022) 181-194.

[29] K. Rajala, J.T. Lehto, E. Sutinen, H. Kautiainen, M. Myllärniemi, T. Saarto, Marked deterioration in the quality of life of patients with idiopathic pulmonary fibrosis during the last two years of life, BMC Pulm Med 18(1) (2018) 172.

For peer review only

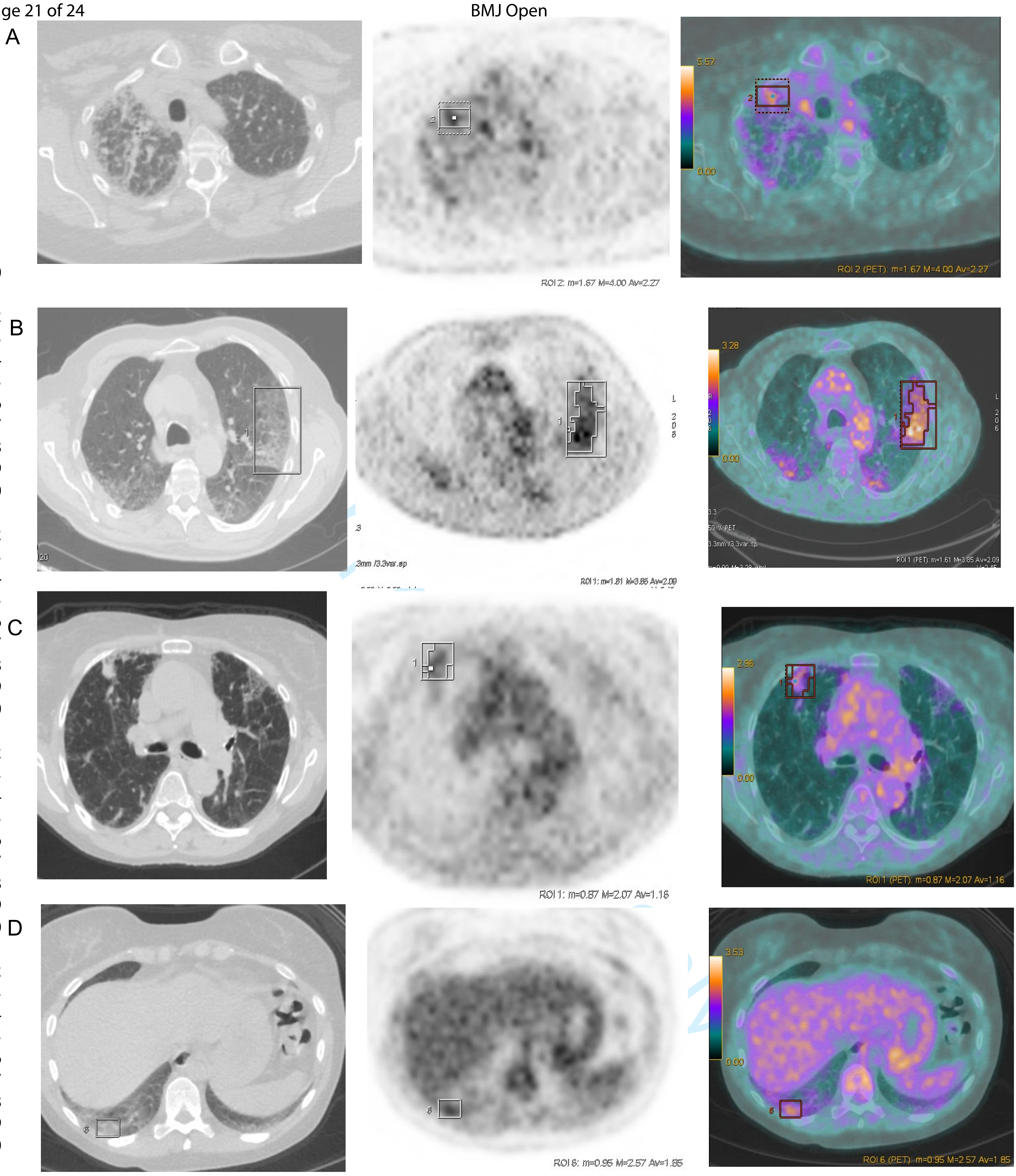

Figure 1: Example PET-CT images from study patients. Images show the helical acquisition CT scan, attenuation-corrected F18 emission and the axially fused PET-CT (L→R). Boxed area on the scans indicates region of SUVmax. **A:** SUVmax 4.1, SGRQ 72.99, **B:** SUVmax 3.9, SGRQ 3.9, **C:** SUVmax 2.1, SGRQ 7.85, **D:** SUVmax 2.68, SGRQ 15.66.

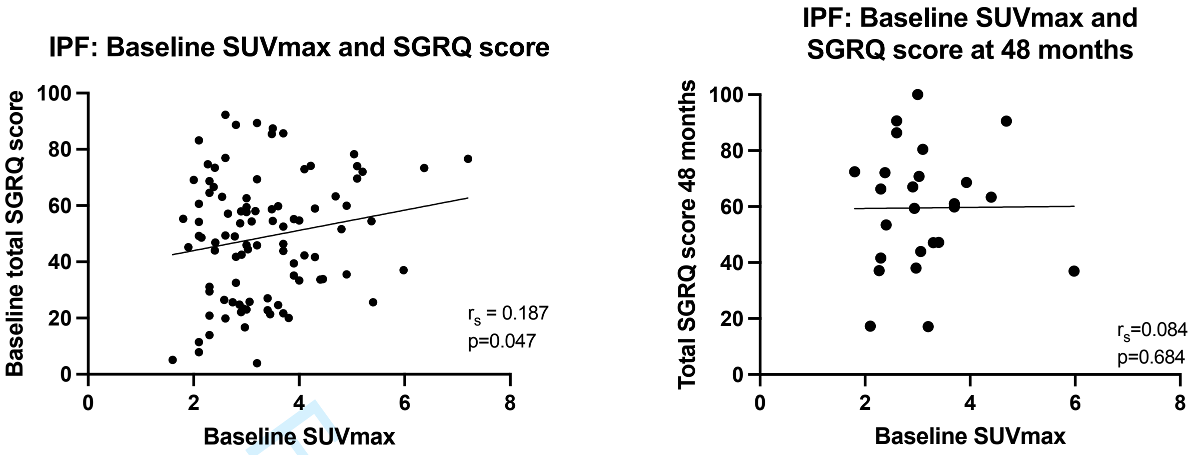

Figure 2: Relationship between baseline SUVmax and SGRQ at (A) baseline and (B) 48 months follow up in the IPF subgroup

For peer review only

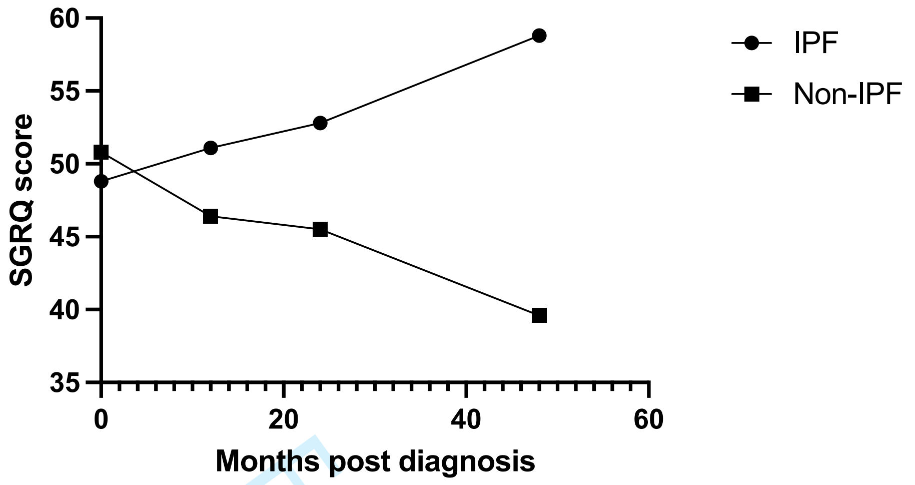

Figure 3: SGRQ by ILD group over 48 month follow up period.

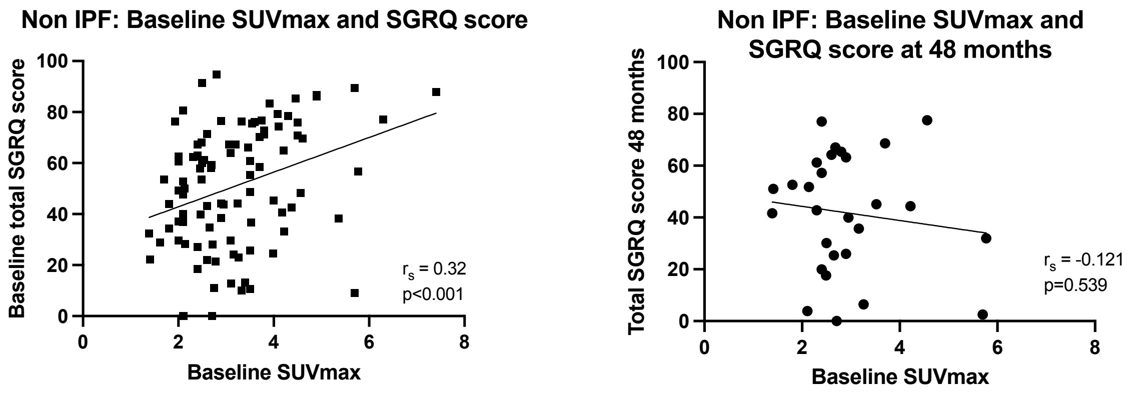

Figure 4: Relationship between baseline SUVmax and SGRQ at (A) baseline and (B) 48 months follow up in the non-IPF subgroup

For peer review only

Supplemental Materials

Table S1: Mean SGRQ scores for the total study population at each time point

	Total	Symptoms	Activity	Impact
**T0**	49.8	54.7	64.1	39.9
**T12**	48.6	51.0	64.7	36.4
**T24**	48.7	50.8	64.2	38.3
**T48**	48.9	52.2	66.2	39.4

Table S2: Total and subscale SGRQ scores at each time point for the IPF subgroup

	Total	Symptoms	Activity	Impact
**T0**	48.8	57.1	63.1	37.8
**T12**	51.1	58.5	66.1	39.3
**T24**	52.8	56.6	67.7	42.8
**T48**	58.8	60.8	72.7	48.7

Table S3: Total and subscale SGRQ scores at each time point for the non-IPF subgroup

	Total	Symptoms	Activity	Impact
**T0**	50.8	52.3	65.1	41.9
**T12**	46.4	44.6	63.3	33.8
**T24**	45.5	46.5	61.6	35.3
**T48**	39.6	43.4	59.1	30.1

For peer review only

BMJ Open

Can FDG-PET/CT imaging be used to predict decline in quality of life in Interstitial Lung Disease? A prospective study of the relationship between FDG uptake and quality of life.

Journal:	*BMJ Open*
Manuscript ID	bmjopen-2023-081103.R1
Article Type:	Original research
Date Submitted by the Author:	23-Feb-2024
Complete List of Authors:	Jordon, Louise Helen; University of Cambridge, ; Cambridge University Hospitals NHS Foundation Trust,<br>Ganeshan, Balaji; UCL Institute of Nuclear Medicine<br>Nadeem, Iftikhar; Cambridge University Hospitals NHS Foundation Trust<br>Hoy, Luke; UCL Institute of Nuclear Medicine<br>Mahdi, Noor; Cambridge University Hospitals NHS Foundation Trust,<br>Porter, Joanna; UCLH, ILD Centre; UCL, Respiratory Medicine<br>Groves, Ashley; UCL Institute of Nuclear Medicine<br>Win, Thida; Lister Hospital
**Primary Subject Heading**:	Respiratory medicine
Secondary Subject Heading:	Radiology and imaging
Keywords:	Interstitial lung disease < THORACIC MEDICINE, NUCLEAR MEDICINE, Quality of Life

SCHOLARONE™
Manuscripts

I, the Submitting Author has the right to grant and does grant on behalf of all authors of the Work (as defined in the below author licence), an exclusive licence and/or a non-exclusive licence for contributions from authors who are: i) UK Crown employees; ii) where BMJ has agreed a CC-BY licence shall apply, and/or iii) in accordance with the terms applicable for US Federal Government officers or employees acting as part of their official duties; on a worldwide, perpetual, irrevocable, royalty-free basis to BMJ Publishing Group Ltd ("BMJ") its licensees and where the relevant Journal is co-owned by BMJ to the co-owners of the Journal, to publish the Work in this journal and any other BMJ products and to exploit all rights, as set out in our licence.

The Submitting Author accepts and understands that any supply made under these terms is made by BMJ to the Submitting Author unless you are acting as an employee on behalf of your employer or a postgraduate student of an affiliated institution which is paying any applicable article publishing charge ("APC") for Open Access articles. Where the Submitting Author wishes to make the Work available on an Open Access basis (and intends to pay the relevant APC), the terms of reuse of such Open Access shall be governed by a Creative Commons licence – details of these licences and which Creative Commons licence will apply to this Work are set out in our licence referred to above.

Other than as permitted in any relevant BMJ Author's Self Archiving Policies, I confirm this Work has not been accepted for publication elsewhere, is not being considered for publication elsewhere and does not duplicate material already published. I confirm all authors consent to publication of this Work and authorise the granting of this licence.

For peer review only

Can FDG-PET/CT imaging be used to predict decline in quality of life in Interstitial Lung Disease? A prospective study of the relationship between FDG uptake and quality of life.

Dr Louise Jordon[1,2] *, Dr Balaji Ganeshan[3], Dr Iftikhar Nadeem[4], Luke Hoy[3], Dr Noor Mahdi[4], Prof Joanna C Porter[5,6], Prof Ashley Groves[3], Prof Thida Win[7]

[1]Department of Respiratory Medicine, Cambridge University Hospitals, Cambridge, CB2 0QQ (UK), [2]Jeffrey-Cheah Biomedical Centre, University of Cambridge, Cambridge, CB2 0AW (UK), [3]Institute of Nuclear Medicine, University College London Hospital (UCLH), London, NW1 2PG (UK), [4]Department of Respiratory Medicine Royal Papworth NHS Foundation Trust, Cambridge, CB2 0AY (UK), [5]Department of Respiratory Medicine, University College London, London, WC1E 6JF (UK), [6]ILD Centre, UCLH, London, WC1E 6JF (UK), [7]Department of Respiratory Medicine, Lister Hospital , Stevenage, SG1 4AB (UK).

*Corresponding author: Dr Louise Jordon, University of Cambridge Department of Medicine, Jeffrey Cheah Biomedical Centre, Puddicombe Way, Cambridge, CB2 0QQ. Email: louise.jordon@nhs.net, 07982713087

For peer review only

ABSTRACT

Background

^{18}F-FDG Positron Emission Tomography (PET) CT imaging has been used in many inflammatory and infectious conditions to differentiate areas of increased metabolic activity. FDG uptake differs between areas of normal lung parenchyma and interstitial lung disease (ILD).

Objectives

In this study we investigated whether FDG-PET/CT parameters were associated with change in quality of life (QoL) in patients with ILD over 4 years of follow-up.

Methods

Patients underwent PET-CT imaging at diagnosis and were followed up with annual QoL assessment using the St George's Respiratory Questionnaire (SGRQ) until death or 4 years of follow-up. Maximum Standard Uptake Value (SUVmax) and Tissue-to-Background Ratio (TBR) were assessed against SGRQ overall and subscale scores.

Results

193 patients (94 Idiopathic Pulmonary Fibrosis; IPF and 99 non-IPF patients underwent baseline FDG-PET/CT imaging and QoL assessment. Weak-to-moderate correlation was observed between baseline SUVmax and SGRQ scores in both ILD subgroups. No relationship was observed between baseline SUVmax or TBR and change in SGRQ scores over 4 years of follow-up. In the IPF subgroup, surviving patients reported a decline in QoL at 4 years post diagnosis whereas an improvement in QoL was seen in surviving non-IPF patients.

Conclusions

Weak-to-moderate positive correlation between baseline SUVmax and SGRQ scores was observed in both ILD groups (IPF:r_s=0.191, p=0.041, non-IPF: r_s=0.320, p=0.001). However, baseline SUVmax and TBR were not associated with change in QoL in patients with IPF and non-IPF ILD over 4 years of follow-up. At 4 years post diagnosis, surviving patients with IPF reported declining QoL whereas improvement was seen in non-IPF patients.

<u>Key words:</u>

Interstitial lung disease, idiopathic pulmonary fibrosis, nuclear medicine, quality of life

<u>Strengths and Limitations:</u>

Strengths:

- Large sample of patients with idiopathic pulmonary fibrosis (IPF) and non-IPF ILD.
- 4 years of quality of life follow up data for patients with good questionnaire return rates in surviving patients

Limitations:

- Treatment and specific ILD subtype (aside from IPF) data not collected

For peer review only

For peer review only - http://bmjopen.bmj.com/site/about/guidelines.xhtml

INTRODUCTION

Interstitial Lung Diseases (ILD) are a heterogenous group of pulmonary conditions characterised by varying degrees of fibrosis and inflammation of the lung parenchyma. ILDs are most frequently diagnosed by a combined clinical and radiological assessment [1]. Of these, Idiopathic Pulmonary Fibrosis (IPF) and Non-Specific Interstitial Pneumonia (NSIP) are the most frequently reported, with progressive fibrosis as the dominant histopathological lesion in IPF, and areas of inflammation and, in some cases, fibrosis occurring in NSIP [2].

Despite anti-fibrotic treatments, the prognosis for IPF is poor, with a mean survival of 2.5-5 years from diagnosis [3]. Quality of life (QoL) in IPF is affected by severe breathlessness and activity impairment [4], which demonstrates a close relationship with changes in lung function [5]. Prognosis and symptom burden in NSIP are much better, with an 86-98% 5-year survival for certain subtypes [6].

At diagnosis and during follow-up, it is difficult to predict prognosis for patients with ILD. When asked, patients have reported outcomes which they value most to be symptom burden, daily functioning and QoL [7]. Presently, decline in Forced Vital Capacity (FVC) is most frequently used to monitor disease progression [8], however baseline FVC is not predictive of change in QoL [5]. Routinely used biomarkers are lacking [9], and so tools to accurately predict QoL deterioration are needed.

Positron Emission Tomography (PET) combined with Computed Tomography (CT) offers a minimally invasive investigation to locate and quantify cellular metabolism. The most frequently used PET tracer is the glucose analogue, fluorodeoxyglucose (FDG). When labelled with the radioisotope 18-Fluorine (^{18}F), it can be used to visualise areas of regional glucose uptake and indicate metabolic activity [10-12].

Tissue metabolic activity on PET-CT is quantified through Standardised Uptake Values (SUVs) which measure the tracer's radioactivity concentration relative to the dose given and the patient's weight [13]. The SUV is most commonly reported as the maximum SUV within the region of interest (SUVmax) [14]. The tissue-to-background ratio (TBR) is a measure of variation of FDG uptake within the lung, where a value close to one indicates relatively uniform uptake [15].

In IPF lung tissue, increased glycolysis has been observed in fibroblasts [16], with increased FDG uptake seen in areas of honeycombing [17, 18]. We have previously shown that FDG uptake on PET/CT is a prognostic biomarker in IPF [19], and showed TBR to be predictive of 3 year mortality. In patients with fibrotic ILD, we have observed that TBR correlates with neoangiogenesis markers [20], and in patients with NSIP we observed that a high pulmonary background signal (SUVmin) negatively correlated with mortality [21]. Others have investigated the utility of FDG uptake in ILD subtype differentiation [22] and in determining disease activity in ILDs associated with rheumatological conditions [23].

For peer review only - http://bmjopen.bmj.com/site/about/guidelines.xhtml

The primary aim of this study was to investigate any relationship between baseline FDG-PET signal (measured by SUVmax, SUVmin and TBR) and QoL (as a self-reported questionnaire) at time of diagnosis and longitudinally over a four year follow up period in patients with ILD, to determine the utility of ^{18}F-FDG PET/CT in ILD QoL prognostication.

For peer review only

For peer review only - http://bmjopen.bmj.com/site/about/guidelines.xhtml

MATERIALS AND METHODS

Study setting and patient selection

This was a prospective, single-centre study from one UK hospital. Patients were recruited from a general respiratory clinic and enrolled for 4 years during the study period from November 2009 to December 2020.

All patients underwent full clinical assessment including multidisciplinary team (MDT) review, baseline pulmonary function tests, and high-resolution CT (HRCT) evaluation. Infection and neoplasia were excluded on clinical and radiological grounds. The diagnosis of IPF was made by MDT review and was based upon HRCT findings of a "usual interstitial pneumonia" (UIP) pattern with no associated cause. If patients did not meet these diagnostic criteria, they were grouped as non-IPF ILD. Written informed consent was obtained for all participants.

Patients had an ^{18}F-FDG PET/CT scan and completed a concurrent baseline QoL questionnaire. They were subsequently followed for 48-months with QoL questionnaires at 12, 24, and 48-month intervals. Patients were excluded if they did not complete a valid QoL questionnaire at baseline, if the SUVmax and TBR could not be calculated or if a diagnosis of IPF or non-IPF ILD could not be reached. A valid questionnaire was one completed in accordance with instructions, with enough questions answered to generate a score.

Baseline demographic and pulmonary function test (PFT) data was also collected. Repeated pulmonary function tests were attempted at 12, 24 and 48 months of follow-up. Baseline ILD treatment data was available for a subset of patients. Baseline treatment was regarded as treatment prescribed within the 12 months up to and including the study commencement date.

In total, 234 patients with ILD were recruited and had ^{18}F-FDG PET/CT scans with quantification of SUVmax, SUVmin and TBR. 41 patients were excluded as they did not complete a valid baseline questionnaire, leaving 193 patients in the study.

Study objective

The primary aim of this study was to investigate the relationship between baseline ^{18}F-FDG PET/CT pulmonary uptake (SUVmax and TBR) and QoL measures, assessed using the St George's Respiratory Questionnaire (SGRQ) [24], at the time of the scan and at 12, 24 and 48 months post baseline.

Secondary objectives included looking for correlation between FDG uptake and QoL subscale scores in the total study population and in IPF and non-IPF groups, and observing QoL trajectory over the study period.

For peer review only - http://bmjopen.bmj.com/site/about/guidelines.xhtml

QoL data collection

The SGRQ is a disease-specific questionnaire designed to measure the impact on overall health, daily life, and perceived well-being in patients with respiratory disease [24, 25]. It has 50 questions divided into three sections covering: respiratory symptoms, activities that cause or are limited by breathlessness and the psycho-social impact of disease. Each question has an empirically derived weight [24], and results in a subsection and overall score out of 100, with higher scores reflecting increased severity. The SGRQ has been well validated in UK and international populations [24, 25].

Baseline SGRQ data was collected when patients attended for their 18F-FDG PET/CT scan (T0), and then at 12, 24 and 48-months later (T12, T24, T48, respectively), by postal questionnaire. QoL trends were calculated by subtracting the baseline QoL score from the score at a subsequent timepoint, thus a positive or negative score indicated a deterioration or improvement respectively.

FDG PET-CT imaging data collection

The 18F-FDG PET/CT was performed as previously described [15].

Patients fasted for 6 hours. Images were acquired 1 hour after an injection of ^{18}F-FDG (200MBq) on a dedicated combined PET/64-detector CT scanner (GE Healthcare). Three sequential scans of the thorax were performed while the patient remained supine on the table with their arms above their head. First, an attenuation-correction CT (CTAC) was performed using 64 3.75 mm detectors, a pitch of 1.5, and a 5-mm collimation (140kVp and 80mA in 0.8s).

Patient position was maintained while a whole-body ^{18}F-FDG PET scan was acquired, covering an area identical to the CTAC. All images were acquired in 2-dimensional mode (8min/bed position). Transaxial emission images of 3.27mm thickness (pixel size 3.9mm) were reconstructed using ordered-subsets expectation maximization with 2 iterations and 28 subsets. The axial field of view was 148.75mm, resulting in 47 slices per bed position. Next, a deep inspiratory HRCT was performed whilst maintaining patient position, using 64 1.25 mm detectors, pitch of 0.53, and 1.25-mm collimation (120kVp and 100mAs).

Image Analysis

PET/CT images were reviewed by 2 combined radiologist/nuclear medicine physicians in consensus, one of whom had a specialist interest in cardiothoracic PET/CT. PET/CT images were loaded onto an Xeleris workstation (GE Healthcare Technology). Both attenuation and non–attenuation-corrected images were reviewed to ensure that areas of higher-density lung were not inducing attenuation-correction artifacts. The area of most intense pulmonary FDG uptake was identified and SUVmax measured. The HRCT parenchymal pattern in the region was also assessed. In addition, SUV_{min} was used to determine the background lung uptake, and in turn calculate TBR.

For peer review only - http://bmjopen.bmj.com/site/about/guidelines.xhtml

Statistical Analysis:

Statistical analyses were performed using SPSS v26 (IBM) and Prism v8 (GraphPad). A two-sided p-value of less than 0.05 indicated a significant relationship. The relationship between FDG-PET/CT uptake and QoL was assessed using non-parametric Spearman's rank correlation.

Patient and public involvement statement:

Whilst patients were not involved directly in the design of this study, the research question and outcome measures were focused upon QoL rather than lung function or other physical parameters in line with patients' greatest priorities.

For peer review only

RESULTS

Total population

Baseline characteristics

In total, 193 patients met the inclusion criteria (131 males, 62 females); average age ± SD: 66.6+/-9 years). **Table 1** shows baseline characteristics

Table 1: Baseline characteristics of the study population

	Data
**Males (%)**	131 (67.8)
**Mean Age (SD)**	66.6 (9)
**Diagnosis: IPF (%)**	94 (48.7)
**NSIP (%)**	78 (40.4)
**Non-NSIP (%)**	21 (10.9)
**Mean baseline FEV1 (SD; range)**	74.9 (18.7; 17-122)
**Mean baseline FVC (SD; range)**	75.8 (17.6; 20-129)
**Mean baseline TLCO (SD; range)**	46.7 (15.5; 11-98)

The mean values for the baseline PET uptake (range in brackets) for the population were: SUVmax 3.25 (1.4-7.4), SUVmin 0.55 (0.16-1.3), and TBR 6.28 (2.4-15.3).

During follow-up of the 193 patients, 21 died by 12 months (10.9%), 42 by 24 months (21.8%) and 120 by 48 months (62.2%).

QoL assessment

QoL data was available for all 193 patients at the time of recruitment (T0), 99 patients at T12 (57.2% surviving patients), 91 patients at T24 (60.3% surviving patients) and 45 patients at T48 (61.6% surviving patients).

Table S1 (supplemental materials) demonstrates the mean QoL scores for the population who returned the SGRQ at the study time points, displayed as a percentage of the total possible score.

There was no significant difference in total SGRQ scores at T0 and T12 ($t=0.44$, $p=0.66$), T24 ($t=0.36$, $p=0.72$) or T48 ($t=0.2$, $p=0.84$).

Relationship between FDG-PET and SGRQ

Example PET-CT images with their corresponding SGRQ scores are shown in **Figure 1A-D.**

At baseline, there was weak positive correlation between SUVmax and total SGRQ score ($r_s=0.229$, $p=0.002$) and the SGRQ Symptom, Activity, and Impact subscales ($r_s=0.211$, 0.178 and 0.209 respectively, $p<0.003$ for all). There was also weak positive correlation between SUVmin and baseline total SGRQ score ($r_s=0.233$, $p=0.001$) and the SGRQ Symptom, Activity, and Impact subscales ($r_s=0.161$, 0.153 and 0.236

For peer review only

respectively, p<0.05 for all). There was no significant relationship between TBR and total (r_s=-0.015, p=0.841) and subscale SGRQ scores.

When change in QoL over time was assessed, there was no significant correlation between change in total SGRQ score and baseline SUVmax at 12 months (r_s = -0.084, p=0.403), 24 months (r_s=0.184, p=0.084) and 48 months (r_s=0.084, p=0.56). Additionally, there was no relationship between change in SGRQ subscale scores at each time point and SUVmax. There was no significant correlation between change in total SGRQ score at 12 months (r_s=-0.017, p=0.863), 24 months (r_s=0.122, p=0.253 and 48 months (r_s=0.140, p=0.332) and baseline SUVmin. We observed no significant correlation between change in total SGRQ score and baseline TBR at T12 (r_s= -0.065, p=0.519), T24 (r_s= -0.014, p=0.893) and T48 (r_s= -0.113, p=0.435), and no relationship between change in SGRQ subscale scores at each time point and TBR.

Relationship between FDG-PET, SGRQ and other parameters
Age at enrolment did not correlate with baseline SUVmax (r_s = -0.103, p=0.158) or baseline TBR (r_s= -0.033, p=0.646).

Baseline FVC and TLCO data were available for 185 and 163 patients respectively. Baseline PET parameters did not correlate with baseline PFT data, nor did baseline PET parameters correlate with change in PFT data after 12, 24 or 48 months of follow up. Further details are included in the supplemental materials (**Table S2**).

ILD treatment data was available for 127 participants at baseline (127/193; 66%). Of these, 84 participants were on no treatment, 35 were on steroid treatment, 9 were on antifibrotic treatments and 10 were on immunosuppressant treatment. 13 patients were on more than one treatment at baseline, 11 of which comprised treatments in different categories. In this subgroup of patients where treatment data was available, type of treatment (or no treatment) at baseline did not affect baseline SUVmax (H(4)=0.88, p=0.83) or SUVmin (H(4)=4.41, p=0.22). There was a significant difference in median TBR between the treatment groups (Median TBR by group: No treatment = 6.0, Steroid = 5.0, Antifibrotic = 5.3, Immunosuppressant = 4.0, H(4)=8.54, p=0.036.

There was weak negative correlation between baseline FVC and baseline TLCO and total baseline SGRQ score (FVC: r_s=-0.285, p<0.001, TLCO: r_s=-0.214,p=0.006). Change in PFT measures did not correlate with change in total SGRQ score over the 48 month follow up period.

IPF- ILD subset
Baseline demographics
There were 94 IPF patients (84 males); average-age ± SD: 69.5+/-8.9 years) in this subgroup.
The mean (range) of baseline PET metrics were: SUVmax 3.4 (1.8-6.0) and TBR 5.4 (3.1-16.6).

For peer review only - http://bmjopen.bmj.com/site/about/guidelines.xhtml

QoL assessment
QoL data was available in all 94 patients at T0. At 12 months 47 patients (50%) returned the SGRQ, 40 (42.5%) at 24 months and 22 (23.4%) at 48 months. During the follow-up period for the 94 patients, 13 died by 12 months (13.8%), 24 by 24 months (25.5%) and 74 by 48 months (78.7%).

Table S3 (supplemental materials) shows the total and subscale SGRQ scores at each time point for the IPF subgroup

When change in QoL was assessed over time, there was no significant difference in total SGRQ at T0 and T12 (t=0.74, p=0.46) or T0 and T24 (t=1.23, p=0.22) but a significant increase in total SGRQ, indicating deteriorating QoL, was seen at 48 months (t=-3.1. p=0.002).

Relationship between SGRQ scores and FDG-PET
At baseline, there was weak positive correlation between total SGRQ score and SUVmax (r_s=0.187, p=0.047; **Figure 2**). There was also weak positive correlation between the Activity SGRQ subscale and SUVmax (r_s= 0191, p=0.039). There was no significant relationship between TBR and total (r_s=0.061, p=0.524) and subscale SGRQ scores, and no significant relationship between SUVmin and total (r_s=0.107, p=0.26) and subscale SGRQ scores.

When change in QoL over time was assessed against pulmonary FDG-PET uptake, there was no significant correlation between change in total SGRQ score and baseline SUVmax at T12 (r_s=0.065, p =0.653), T24 (r_s=0.017, p=0.913) and T48 (r_s=0.084, p=0.684; Figure 1). Additionally, there was no relationship between change in SGRQ subscale scores at each time point and SUVmax, and no relationship between total or subscale SGRQ scores and SUVmin.

We also observed no significant correlation between change in total SGRQ score and baseline TBR at T12 (r_s=0.035, p=0.807), T24 (r_s= -0.122, p=0.419) and T48 (r_s=0.006, p=0.978, and no relationship between change in SGRQ subscale scores at each time point and TBR.

For peer review only

Non-IPF subset
Baseline demographics
There were 99 Non-IPF ILD patients (45 males); mean age ±SD: 64.6+/-10.1 years). The mean (range) of baseline PET uptake metrics were: SUVmax 3.1 (1.4-6.4) and TBR 6.8 (2.9-13.6).

QoL assessment
QoL data was available in all 99 patients at the time of recruitment (T0). 52 patients (52.5%) returned the QoL questionnaire at 12 months, 51 (54.3%) at 24 months and

For peer review only - http://bmjopen.bmj.com/site/about/guidelines.xhtml

23 (23.2%) at 48 months. Of the 99 patients, 8 died by 12 months (8.1%), 18 by 24 months (18.1%) and 46 by 48 months (46.5%) of follow up.

Table S4 (supplemental materials) shows the total and subscale SGRQ scores at each time point for the non-IPF subgroup

When change in QoL was assessed over time, there was no significant difference in total SGRQ at T0 and T12 (t=1.14, p=0.26) or T0 and T24 (t=1.23, p=0.22) but a significant decrease in total SGRQ, indicating improved QOL, was seen at 48 months (t=2.04. p=0.05).

In comparison with total SGRQ scores from the IPF subgroup, there was no significant difference at T0 (t=0.614, p=0.54), T12 (t=1.09, p=0.28) or T24 (t=1.43, p=0.16). Scores in the non-IPF group were significantly lower than those of the IPF group at T48 (t=-3.15, p=0.003). Comparison of overall SGRQ scores between the IPF and non-IPF subgroups is shown in **Figure 3.**

Relationship between SGRQ scores and FDG-PET

At baseline there was moderately positive correlation with total SGRQ score and SUVmax (r_s=0.320, p=0.001; **Figure 4**). There was also positive correlation between the baseline Symptoms, Activity and Impact subscale scores and baseline SUVmax (r_s= 0.287, 0.242 and 0.314, p<0.008 respectively). As baseline there was moderately positive correlation between total SGRQ and SUVmin (r_s=0.390, p=0.001). There was also positive correlation between the baseline Symptoms, Activity and Impact subscale scores and baseline SUVmin (r_s= 0.230, 0.212 and 0.366, p<0.01 respectively). There was no significant relationship between TBR and total (r_s=-0.085, p=0.379) and subscale SGRQ scores.

When change in QoL over time was assessed against pulmonary FDG PET uptake, there was no significant correlation between change in total SGRQ score and baseline SUVmax at T12 (r_s= -0.126, p=0.347), T24 (r_s=0.188, p=0.188) and T48 (r_s= -0.121, p=0.539; Figure 3). Additionally, there was no relationship between change in SGRQ subscale scores at each time point and baseline SUVmax, and no relationship between change in total or subscale SGRQ scores and SUVmin over time.

We also observed no significant correlation between change in total SGRQ score and baseline TBR at T12 (r_s= -0.051, p=0.702), T24 (r_s=0.075, p=0.603) and T48 (r_s=0.083, p=0.676, and no relationship between change in SGRQ subscale scores at each time point and TBR.

For peer review only - http://bmjopen.bmj.com/site/about/guidelines.xhtml

DISCUSSION

The aim of this study was to investigate the relationship between ^{18}FDG-PET/CT metrics and QoL in patients with IPF and non-IPF ILD to determine if ^{18}FDG-PET/CT could be used in ILD QoL prognostication. To our knowledge, this study is the first and largest to date investigating this relationship.

Relationship between FDG-PET/CT and QoL

Our study showed that there was weak association between baseline SUVmax and QoL measured by the SGRQ. This may indicate that the metabolic activity of ILD may relate to a patient's symptoms and QoL. However, in both IPF and non-IPF subgroups, we observed no significant relationship between baseline SUVmax or TBR and change in QoL over the 48-month follow-up period. As such, these baseline PET measurements do not appear to be associated with patient-reported change in QoL for either ILD subgroup.

Work from our group has previously shown higher TBR to be predictive of survival in patients with IPF [19]. More recently we have also shown TBR to correlate with biomarkers of new vessel angiogenesis, which itself is predictive of poorer survival [26]. In 27 patients with IPF, SUVmax was shown to be predictive of FVC, TLCO and physiological decline at 12 months [18]. A larger study of 89 patients reported baseline SUVmax to be predictive of TLCO decline at 6 months, but no relationship was observed with survival outcomes [27].

However, existing literature on FDG-PET/CT as a prognostic tool in IPF is mixed. Groves *et al* [17] observed a relationship between baseline SUVmax and baseline global health and lung function in 18 patients with IPF, but these measures were not predictive of decline at 6 months. This concurs with our findings of correlation between baseline SUVmax and QoL measures in IPF patients. When evaluating the role of 18F-FDG PET/CT in predicting treatment response in IPF, Bondue *et al* found SUVmax to be predictive of pirfenidone response in a mouse bleomycin model, but not in 25 patients with IPF, and nor was it predictive of disease progression during the 3-month study period in the patient population [28].

Small previous studies have shown FDG uptake distribution in NSIP, but not SUVmax to be predictive of response to treatment [29]. In patients with advanced systemic sclerosis associated ILD, ^{18}F-FDG PET/CT was unable to differentiate between areas of active inflammation (ground glass opacities on HRCT) and areas of fibrosis, but could differentiate between normal lung parenchyma and ILD changes.

To our knowledge, this is the first study evaluating the relationship between patient reported QoL and SUVmax and TBR. Whilst some studies have shown a relationship between these parameters and lung function or survival, we may not have seen a relationship between them and QoL trajectory because QoL has many more contributing factors. Furthermore, the drop out in questionnaire returns over our study period is due to patient death, and hence only patients with a milder disease trajectory were evaluated at our 48-month time point. Additionally, whilst baseline FEV1 and FVC measures of our patients were similar to those in other studies [18], our patients were enrolled from diagnosis whereas other studies enrolled subjects at later disease stages. Finally, studies evaluating PET-CT as a prognostic tool have focused on

For peer review only - http://bmjopen.bmj.com/site/about/guidelines.xhtml
For peer review only

different PET parameters such as FDG uptake pattern and SUVmean, and hence using these may have led to an observed relationship with QoL.

Combining this study with prior work [5, 17, 19], there is presently insufficient evidence to support using baseline FVC [5] or SUVmax/TBR to predict symptomatic decline in ILD [17], but these can be used to predict survival outcomes in IPF [19].

QoL trajectory in patients with IPF and non-IPF ILD

The trajectory of patients' QoL over the follow-up period was evaluated as one of our secondary outcomes. Better QoL was observed in the non-IPF subgroup compared to the IPF group at all points from 12-48 months follow-up In the IPF subgroup, QoL deterioration was observed at the 48 month time point, but not prior to this. Notably, 78% of the initial IPF cohort had died at 48 months. For the non-IPF group, there was no difference in QoL measures at 12 and 24 months compared with baseline, but a slight improvement seen at 48 months, by which point 46% of the cohort had died. Thus, patients with IPF who survived to 48 months post diagnosis noted a decline in their QoL at this stage, whereas surviving non-IPF ILD patients began to improve at this stage. This enhances our knowledge of known prognostic indicators for different types of ILD. Improving QoL over the longer term in non-IPF patients is an encouraging finding. The non-IPF ILD subgroup represent a clinically heterogenous group. The observation of improvement in QoL in surviving patients is likely due to effective treatment options for certain NSIP subtypes.

Notably, surviving IPF patients did not note a decline in their QoL until 48 months post diagnosis. This may reflect a milder disease phenotype in the surviving patients, or signal an abrupt symptomatic decline in patients with IPF in the couple of years before they die. A large registry study of 277 IPF patients observed SGRQ-measured QoL was static for 12 months and then declined in patients a mean of three years post IPF diagnosis [30]. In particular, QoL has been shown to be very low in the two years prior to death in IPF patients [31]. Our study supports this existing work, and reinforces the need for palliative care input alongside other treatments for IPF patients at this stage of disease.

Limitations and future work

This study benefits from recruiting a large number of patients who have undergone PET-CT imaging and been followed up for 4 years. Nonetheless, there are some limitations. After baseline QoL assessment, QoL data was only available for approximately 60% of surviving patients at each subsequent time point, which could introduce nonresponse bias. Furthermore, data was not collected on specific non-IPF ILD subtypes and as such we cannot draw conclusions on specific ILDs and the effect of ^{18}F-FDG PET/CT as a prognostic marker.

Future work could focus upon PET/CT measures and QoL in specific ILD subgroups, particularly those with an inflammatory phenotype, and could explore the role of PET-CT in treatment response prediction.

For peer review only - http://bmjopen.bmj.com/site/about/guidelines.xhtml

CONCLUSIONS

^{18}F-FDG PET/CT measure SUVmax demonstrated weak-to-moderate correlation with QoL at baseline, but baseline SUVmax and TBR were not associated with change in QoL in patients with IPF and non-IPF ILD. At present, there is insufficient evidence to support the role of ^{18}F-FDG-PET/CT in QoL prediction in ILD. Further prospective studies of ^{18}F-FDG-PET/CT involving treatment data, QoL and other imaging parameters may be beneficial to identify non-invasive disease markers.

At 4 years post IPF diagnosis, surviving patients reported declining QoL, whereas surviving non-IPF ILD patients reported an improvement in their QoL, potentially reflecting the beneficial effect of treatment.

For peer review only

FIGURE CAPTIONS

Figure 1: Example PET-CT images from study patients. Images show the helical acquisition CT scan, attenuation-corrected F18 emission and the axially fused PET-CT (L→R). Boxed area on the scans indicates region of SUVmax. **A:** SUVmax 4.1, SGRQ 72.99, **B:** SUVmax 3.9, SGRQ 58.83, **C:** SUVmax 2.1, SGRQ 7.85, **D:** SUVmax 2.68, SGRQ 15.66.

Figure 2: Relationship between baseline SUVmax and SGRQ at (A) baseline and (B) 48 months follow up in the IPF subgroup

Figure 3: SGRQ by ILD group over 48 month follow up period.

Figure 4: Relationship between baseline SUVmax and SGRQ at (A) baseline and (B) 48 months follow up in the non-IPF subgroup

For peer review only

For peer review only - http://bmjopen.bmj.com/site/about/guidelines.xhtml

ACKNOWLEDGEMENTS

This work was presented at the 2022 European Respiratory Society International Congress in the ILD poster session. *Eur Respir J* 2022; 60: Suppl. 66, 2473. https://doi.org/10.1183/13993003.congress-2022.2473

CONFLICT OF INTEREST STATEMENT

The authors report no conflicts of interest to declare.

FUNDING SOURCES

This study was undertaken at UCLH/UCL who received a proportion of funding from the Department of Health's NIHR Biomedical Research Centre's funding scheme.

STATEMENT OF ETHICS

This study was approved by the Joint UCL/UCLH Committees on the Ethics of Human Research (Committee A)

REC reference number 06/Q0505/22

DATA AVAILABILITY

Data from this study are available upon reasonable request. This comprises deidentified patient demographic and quality of life data, and calculated PET parameters. Images from which PET parameters were calculated may also be made available upon reasonable request.

AUTHOR CONTRIBUTIONS

LJ contributed to the data analysis (statistical), data visualization and drafted the original manuscript. BG contributed to data curation, data analysis (imaging and statistical), investigation, review and editing. LH contributed to data curation, investigation, visualization, review and editing. IN contributed to review and editing. NM contributed to review and editing. AG contributed to conceptualization, funding acquisition, methodology, visualization, review and editing. JP contributed to conceptualization, funding acquisition, methodology, data curation, review and editing. TW contributed to conceptualization, methodology, funding acquisition, project administration, supervision, review and editing. LJ is the submitting and corresponding author.

For peer review only

REFERENCES

[1] K.M. Antoniou, G.A. Margaritopoulos, S. Tomassetti, F. Bonella, U. Costabel, V. Poletti, Interstitial lung disease, Eur Respir Rev 23(131) (2014) 40-54.

[2] R. du Bois, T.E. King, Challenges in pulmonary fibrosis x 5: the NSIP/UIP debate, Thorax 62(11) (2007) 1008-12.

[3] H. Fujimoto, T. Kobayashi, A. Azuma, Idiopathic Pulmonary Fibrosis: Treatment and Prognosis, Clin Med Insights Circ Respir Pulm Med 9(Suppl 1) (2015) 179-185.

[4] J. De Vries, B.L. Kessels, M. Drent, Quality of life of idiopathic pulmonary fibrosis patients, Eur Respir J 17(5) (2001) 954-61.

[5] M. Kreuter, J. Swigris, D. Pittrow, S. Geier, J. Klotsche, A. Prasse, H. Wirtz, D. Koschel, S. Andreas, M. Claussen, C. Grohé, H. Wilkens, L. Hagmeyer, D. Skowasch, J.F. Meyer, J. Kirschner, S. Gläser, N. Kahn, T. Welte, C. Neurohr, M. Schwaiblmair, M. Held, T. Bahmer, T. Oqueka, M. Frankenberger, J. Behr, The clinical course of idiopathic pulmonary fibrosis and its association to quality of life over time: longitudinal data from the INSIGHTS-IPF registry, Respir Res 20(1) (2019) 59.

[6] W.D. Travis, K. Matsui, J. Moss, V.J. Ferrans, Idiopathic nonspecific interstitial pneumonia: prognostic significance of cellular and fibrosing patterns: survival comparison with usual interstitial pneumonia and desquamative interstitial pneumonia, Am J Surg Pathol 24(1) (2000) 19-33.

[7] K.I. Aronson, S.K. Danoff, A.M. Russell, C.J. Ryerson, A. Suzuki, M.S. Wijsenbeek, S. Bajwah, P. Bianchi, T.J. Corte, J.S. Lee, K.O. Lindell, T.M. Maher, F.J. Martinez, P.M. Meek, G. Raghu, G. Rouland, R. Rudell, M.M. Safford, J.S. Sheth, J.J. Swigris, Patient-centered Outcomes Research in Interstitial Lung Disease: An Official American Thoracic Society Research Statement, Am J Respir Crit Care Med 204(2) (2021) e3-e23.

[8] R.M. du Bois, D. Weycker, C. Albera, W.Z. Bradford, U. Costabel, A. Kartashov, T.E. King, L. Lancaster, P.W. Noble, S.A. Sahn, M. Thomeer, D. Valeyre, A.U. Wells, Forced vital capacity in patients with idiopathic pulmonary fibrosis: test properties and minimal clinically important difference, Am J Respir Crit Care Med 184(12) (2011) 1382-9.

[9] S. Alqalyoobi, A. Adegunsoye, A. Linderholm, C. Hrusch, C. Cutting, S.F. Ma, A. Sperling, I. Noth, M.E. Strek, J.M. Oldham, Circulating Plasma Biomarkers of Progressive Interstitial Lung Disease, Am J Respir Crit Care Med 201(2) (2020) 250-253.

[10] G.I. Bell, C.F. Burant, J. Takeda, G.W. Gould, Structure and function of mammalian facilitative sugar transporters, J Biol Chem 268(26) (1993) 19161-4.

[11] F.J. Gallagher BM , Gutterson NI , et al, Metabolic trapping as a principle of radiopharmaceutical design: some factors responsible for the biodistribution of [18 F] 2-deoxy-2-fluoro- d -glucose . Journal of Nuclear Medicine 19 (1978) 1154-1161.

For peer review only - http://bmjopen.bmj.com/site/about/guidelines.xhtml

[12] E.K. Pauwels, M.J. Ribeiro, J.H. Stoot, V.R. McCready, M. Bourguignon, B. Mazière, FDG accumulation and tumor biology, Nucl Med Biol 25(4) (1998) 317-22.

[13] P.E. Kinahan, J.W. Fletcher, Positron emission tomography-computed tomography standardized uptake values in clinical practice and assessing response to therapy, Semin Ultrasound CT MR 31(6) (2010) 496-505.

[14] M.C. Adams, T.G. Turkington, J.M. Wilson, T.Z. Wong, A systematic review of the factors affecting accuracy of SUV measurements, AJR Am J Roentgenol 195(2) (2010) 310-20.

[15] T. Win, N.J. Screaton, J.C. Porter, B. Ganeshan, T.M. Maher, F. Fraioli, R. Endozo, R.I. Shortman, L. Hurrell, B.F. Holman, K. Thielemans, A. Rashidnasab, B.F. Hutton, P.T. Lukey, A. Flynn, P.J. Ell, A.M. Groves, Pulmonary 18 F-FDG uptake helps refine current risk stratification in idiopathic pulmonary fibrosis (IPF), Eur J Nucl Med Mol Imaging 45(5) (2018) 806-815.

[16] N. Xie, Z. Tan, S. Banerjee, H. Cui, J. Ge, R.M. Liu, K. Bernard, V.J. Thannickal, G. Liu, Glycolytic Reprogramming in Myofibroblast Differentiation and Lung Fibrosis, Am J Respir Crit Care Med 192(12) (2015) 1462-74.

[17] A.M. Groves, T. Win, N.J. Screaton, M. Berovic, R. Endozo, H. Booth, I. Kayani, L.J. Menezes, J.C. Dickson, P.J. Ell, Idiopathic pulmonary fibrosis and diffuse parenchymal lung disease: implications from initial experience with 18F-FDG PET/CT, J Nucl Med 50(4) (2009) 538-45.

[18] A. Justet, A. Laurent-Bellue, G. Thabut, A. Dieudonné, M.P. Debray, R. Borie, M. Aubier, R. Lebtahi, B. Crestani, [18 F]FDG PET/CT predicts progression-free survival in patients with idiopathic pulmonary fibrosis, Respir Res 18(1) (2017) 74.

[19] T. Win, N.J. Screaton, J.C. Porter, B. Ganeshan, T.M. Maher, F. Fraioli, R. Endozo, R.I. Shortman, L. Hurrell, B.F. Holman, K. Thielemans, A. Rashidnasab, B.F. Hutton, P.T. Lukey, A. Flynn, P.J. Ell, A.M. Groves, Pulmonary 18F-FDG uptake helps refine current risk stratification in idiopathic pulmonary fibrosis (IPF), European Journal of Nuclear Medicine and Molecular Imaging 45(5) (2018) 808-818.

[20] J. Porter, B. Ganeshan, Win T, F. Fraioli, S. Khan, M. Rodrigues-Justo, R. Endozo, R. Shortman, T. Maher, A. Groves, 18F-FDG PET signal correlates with markers of neo-angiogensis in paitnets with fibrotic interstitial lung disease who underwent lung biopsy: implication for the use of PET/CT in diffuse lung diseases. , Research Square [Preprint] (2022).

[21] H. Torlot, T. Win, B. Ganeshan, N. Screaton, T. Maher, F. Fraiolo, R. Endozo, R. Shortman, A. Groves, J. Porter, S. Ratnakumar, Pulmonary 18F-FDG uptake refines current risk stratification in non-specific interstitial pneumonitis (NSIP) and predicts response to treatment., European Respiratory Journal 62 (2023).

[22] S. Nusair, R. Rubinstein, N.M. Freedman, G. Amir, N.R. Bogot, U. Izhar, R. Breuer, Positron emission tomography in interstitial lung disease, Respirology 12(6) (2007) 843-7.

For peer review only - http://bmjopen.bmj.com/site/about/guidelines.xhtml

[23] D.M. Peelen, B.G.J.C. Zwezerijnen, E.J. Nossent, L.J. Meijboom, O.S. Hoekstra, C.J. Van der Laken, A.E. Voskuyl, The quantitative assessment of interstitial lung disease with positron emission tomography scanning in systemic sclerosis patients, Rheumatology (Oxford) 59(6) (2020) 1407-1415.

[24] P.W. Jones, F.H. Quirk, C.M. Baveystock, The St George's Respiratory Questionnaire, Respir Med 85 Suppl B (1991) 25-31; discussion 33-7.

[25] M. Ferrer, C. Villasante, J. Alonso, V. Sobradillo, R. Gabriel, G. Vilagut, J.F. Masa, J.L. Viejo, C.A. Jiménez-Ruiz, M. Miravitlles, Interpretation of quality of life scores from the St George's Respiratory Questionnaire, Eur Respir J 19(3) (2002) 405-13.

[26] J. Porter, B. Ganeshan, T. Win, F. Fraioli, S. Khan, M. Rodriguez-Justo, R. Endozo, R. Shortman, T. Maher, A. Groves, [18 F]FDG PET signal correlates with markers of neo-angiogenesis in patients with fibrotic interstitial lung disease who underwent lung biopsy: Implication for the use of PET/CT in diffuse lung diseases, Preprint: Research Square (2022).

[27] Hee-Young Yoon, Suk Hyun Lee, Sejin Ha , Jin-Sook Ryu , J.W. Song, The Value of 18 F-FDG PET/CT in Evaluating Disease Severity and Prognosis in Idiopathic Pulmonary Fibrosis Patients, 2021, p. 257.

[28] B. Bondue, A. Castiaux, G. Van Simaeys, C. Mathey, F. Sherer, D. Egrise, S. Lacroix, F. Huaux, G. Doumont, S. Goldman, Absence of early metabolic response assessed by 18F-FDG PET/CT after initiation of antifibrotic drugs in IPF patients, Respir Res 20(1) (2019) 10.

[29] V. Jacquelin, A. Mekinian, P.Y. Brillet, H. Nunes, O. Fain, D. Valeyre, M. Soussan, FDG-PET/CT in the prediction of pulmonary function improvement in nonspecific interstitial pneumonia. A Pilot Study, Eur J Radiol 85(12) (2016) 2200-2205.

[30] W.A. Wuyts, C. Dahlqvist, H. Slabbynck, M. Schlesser, N. Gusbin, C. Compere, S. Maddens, S. Rizzo, K.U. Kirchgaessler, K. Bartley, B. Bondue, Quality of Life and Healthcare Resource Use in a Real-world Patient Population with Idiopathic Pulmonary Fibrosis: The PROOF Registry, Pulm Ther 8(2) (2022) 181-194.

[31] K. Rajala, J.T. Lehto, E. Sutinen, H. Kautiainen, M. Myllärniemi, T. Saarto, Marked deterioration in the quality of life of patients with idiopathic pulmonary fibrosis during the last two years of life, BMC Pulm Med 18(1) (2018) 172.

For peer review only - http://bmjopen.bmj.com/site/about/guidelines.xhtml

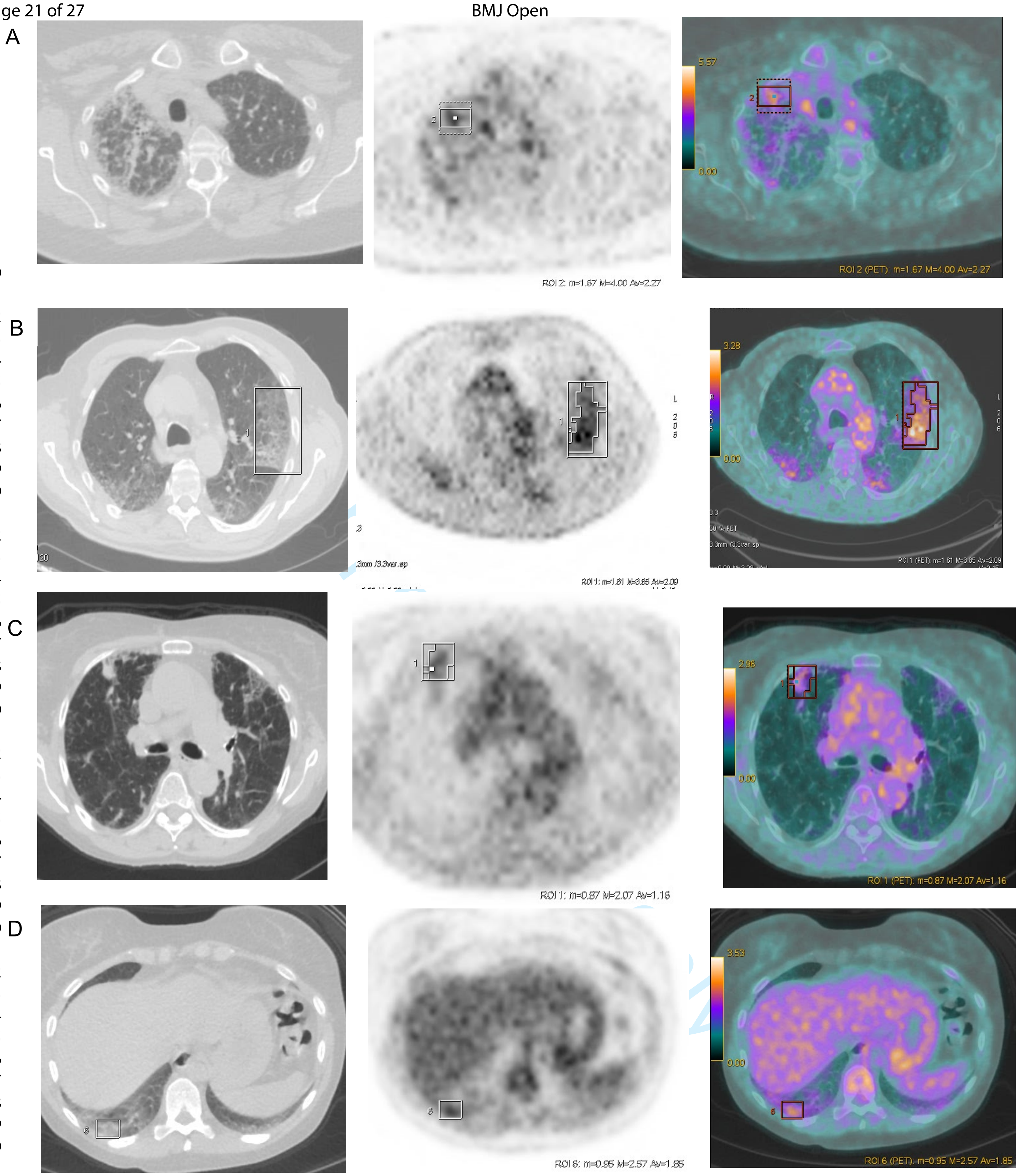

Figure 1: Example PET-CT images from study patients. Images show the helical acquisition CT scan, attenuation-corrected F18 emission and the axially fused PET-CT (L→R). Boxed area on the scans indicates region of SUVmax. **A:** SUVmax 4.1, SGRQ 72.99, **B:** SUVmax 3.9, SGRQ 3.9, **C:** SUVmax 2.1, SGRQ 7.85, **D:** SUVmax 2.68, SGRQ 15.66.

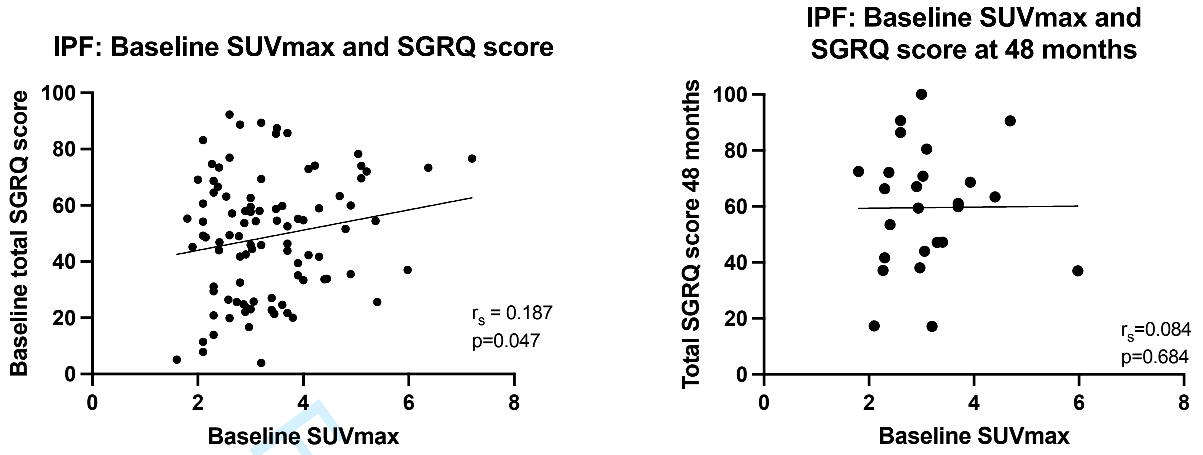

Figure 2: Relationship between baseline SUVmax and SGRQ at (A) baseline and (B) 48 months follow up in the IPF subgroup

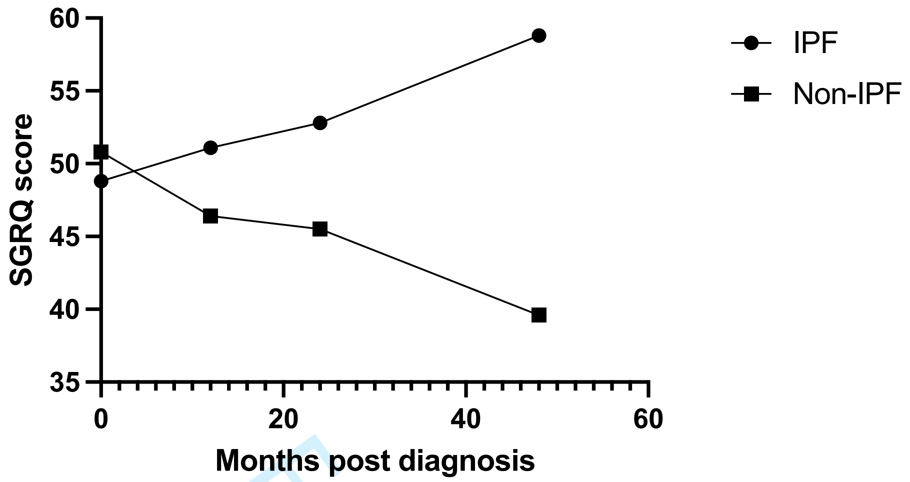

Figure 3: SGRQ by ILD group over 48 month follow up period.

For peer review only

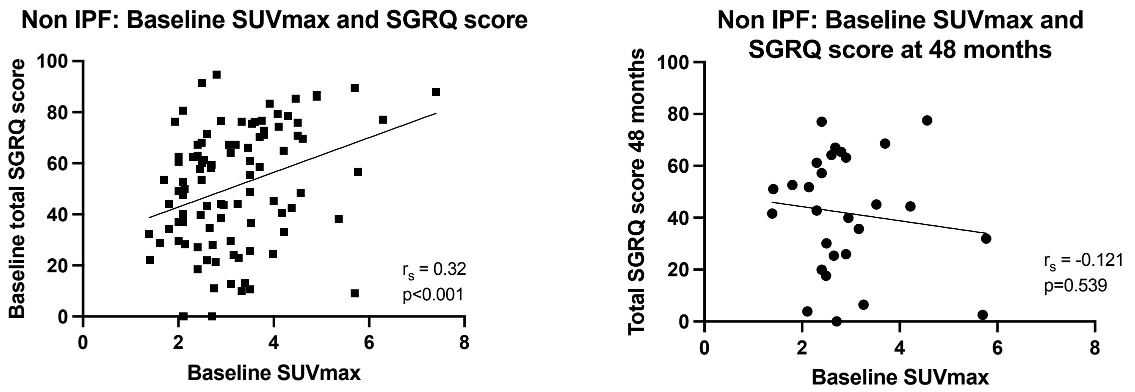

Figure 4: Relationship between baseline SUVmax and SGRQ at (A) baseline and (B) 48 months follow up in the non-IPF subgroup

For peer review only

Supplemental Materials

Table S1: Mean SGRQ scores for the total study population at each time point

	Total	Symptoms	Activity	Impact
**T0**	49.8	54.7	64.1	39.9
**T12**	48.6	51.0	64.7	36.4
**T24**	48.7	50.8	64.2	38.3
**T48**	48.9	52.2	66.2	39.4

Table S2: Correlation between change in PFTs from baseline and baseline FDG-PET parameters

**Correlation between change in FVC from baseline and SUVmax (number of participants)**		
	**Correlation Coefficient (r_s)**	**P value**
**Baseline** (185)	-0.176	0.017
**12 months** (119)	0.010	0.912
**24 months** (73)	0.144	0.225
**48 months** (15)	0.220	0.427
**Correlation between change in TLCO from baseline and SUVmax (number of participants)**		
	**Correlation Coefficient (r_s)**	**P value**
**Baseline** (163)	-0.158	0.043
**12 months** (90)	0.072	0.503
**24 months** (52)	0.030	0.831
**48 months** (10)	0.185	0.604
**Correlation between change in FVC from baseline and TBR (number of participants)**		
	**Correlation Coefficient (r_s)**	**P value**
**Baseline** (185)	-0.003	0.967
**12 months** (119)	-0.101	0.273
**24 months** (73)	0.027	0.818
**48 months** (15)	-0.143	0.611
**Correlation between change in TLCO from baseline and TBR (number of participants)**		
	**Correlation Coefficient (r_s)**	**P value**
**Baseline** (163)	-0.036	0.648
**12 months** (90)	-0.052	0.625
**24 months** (52)	-0.046	0.748
**48 months** (10)	-0.098	0.789

Table S3: Total and subscale SGRQ scores at each time point for the IPF subgroup

	Total	Symptoms	Activity	Impact
**T0**	48.8	57.1	63.1	37.8
**T12**	51.1	58.5	66.1	39.3
**T24**	52.8	56.6	67.7	42.8
**T48**	58.8	60.8	72.7	48.7

For peer review only

Table S4: Total and subscale SGRQ scores at each time point for the non-IPF subgroup

	Total	Symptoms	Activity	Impact
**T0**	50.8	52.3	65.1	41.9
**T12**	46.4	44.6	63.3	33.8
**T24**	45.5	46.5	61.6	35.3
**T48**	39.6	43.4	59.1	30.1

For peer review only

STROBE Statement—Checklist of items that should be included in reports of ***cohort studies***

	Item No	Recommendation	Page No
**Title and abstract**	1	(*a*) Indicate the study's design with a commonly used term in the title or the abstract<br>(*b*) Provide in the abstract an informative and balanced summary of what was done and what was found	1
**Introduction**			
Background/rationale	2	Explain the scientific background and rationale for the investigation being reported	2
Objectives	3	State specific objectives, including any prespecified hypotheses	2-4
**Methods**			
Study design	4	Present key elements of study design early in the paper	4-5
Setting	5	Describe the setting, locations, and relevant dates, including periods of recruitment, exposure, follow-up, and data collection	4-5
Participants	6	(*a*) Give the eligibility criteria, and the sources and methods of selection of participants. Describe methods of follow-up<br>(*b*) For matched studies, give matching criteria and number of exposed and unexposed	4
Variables	7	Clearly define all outcomes, exposures, predictors, potential confounders, and effect modifiers. Give diagnostic criteria, if applicable	4,5
Data sources/ measurement	8*	For each variable of interest, give sources of data and details of methods of assessment (measurement). Describe comparability of assessment methods if there is more than one group	4,5
Bias	9	Describe any efforts to address potential sources of bias	5-6
Study size	10	Explain how the study size was arrived at	
Quantitative variables	11	Explain how quantitative variables were handled in the analyses. If applicable, describe which groupings were chosen and why	5-6
Statistical methods	12	(*a*) Describe all statistical methods, including those used to control for confounding<br>(*b*) Describe any methods used to examine subgroups and interactions<br>(*c*) Explain how missing data were addressed<br>(*d*) If applicable, explain how loss to follow-up was addressed<br>(*e*) Describe any sensitivity analyses	5-6
**Results**			
Participants	13*	(a) Report numbers of individuals at each stage of study—eg numbers potentially eligible, examined for eligibility, confirmed eligible, included in the study, completing follow-up, and analysed<br>(b) Give reasons for non-participation at each stage<br>(c) Consider use of a flow diagram	7
Descriptive data	14*	(a) Give characteristics of study participants (eg demographic, clinical, social) and information on exposures and potential confounders<br>(b) Indicate number of participants with missing data for each variable of interest<br>(c) Summarise follow-up time (eg, average and total amount)	7-9
Outcome data	15*	Report numbers of outcome events or summary measures over time	7-9

For peer review only

For peer review only - http://bmjopen.bmj.com/site/about/guidelines.xhtml

Main results	16	(*a*) Give unadjusted estimates and, if applicable, confounder-adjusted estimates and their precision (eg, 95% confidence interval). Make clear which confounders were adjusted for and why they were included<br>(*b*) Report category boundaries when continuous variables were categorized<br>(*c*) If relevant, consider translating estimates of relative risk into absolute risk for a meaningful time period	7-9
Other analyses	17	Report other analyses done—eg analyses of subgroups and interactions, and sensitivity analyses	7-10
**Discussion**			
Key results	18	Summarise key results with reference to study objectives	11
Limitations	19	Discuss limitations of the study, taking into account sources of potential bias or imprecision. Discuss both direction and magnitude of any potential bias	12
Interpretation	20	Give a cautious overall interpretation of results considering objectives, limitations, multiplicity of analyses, results from similar studies, and other relevant evidence	11-12
Generalisability	21	Discuss the generalisability (external validity) of the study results	11-13
**Other information**			
Funding	22	Give the source of funding and the role of the funders for the present study and, if applicable, for the original study on which the present article is based	15

*Give information separately for exposed and unexposed groups.

Note: An Explanation and Elaboration article discusses each checklist item and gives methodological background and published examples of transparent reporting. The STROBE checklist is best used in conjunction with this article (freely available on the Web sites of PLoS Medicine at http://www.plosmedicine.org/, Annals of Internal Medicine at http://www.annals.org/, and Epidemiology at http://www.epidem.com/). Information on the STROBE Initiative is available at http://www.strobe-statement.org.

For peer review only

BMJ Open

Can FDG-PET/CT imaging be used to predict decline in quality of life in Interstitial Lung Disease? A prospective study of the relationship between FDG uptake and quality of life in a UK outpatient setting.

Journal:	*BMJ Open*
Manuscript ID	bmjopen-2023-081103.R2
Article Type:	Original research
Date Submitted by the Author:	23-Apr-2024

Complete List of Authors:	Jordon, Louise Helen; University of Cambridge, ; Cambridge University Hospitals NHS Foundation Trust,<br>Ganeshan, Balaji; UCL Institute of Nuclear Medicine<br>Nadeem, Iftikhar; Cambridge University Hospitals NHS Foundation Trust<br>Hoy, Luke; UCL Institute of Nuclear Medicine<br>Mahdi, Noor; Cambridge University Hospitals NHS Foundation Trust,<br>Porter, Joanna; UCLH, ILD Centre; UCL, Respiratory Medicine<br>Groves, Ashley; UCL Institute of Nuclear Medicine<br>Win, Thida; Lister Hospital

**Primary Subject Heading**:	Respiratory medicine
Secondary Subject Heading:	Radiology and imaging
Keywords:	Interstitial lung disease < THORACIC MEDICINE, NUCLEAR MEDICINE, Quality of Life

SCHOLARONE™
Manuscripts

I, the Submitting Author has the right to grant and does grant on behalf of all authors of the Work (as defined in the below author licence), an exclusive licence and/or a non-exclusive licence for contributions from authors who are: i) UK Crown employees; ii) where BMJ has agreed a CC-BY licence shall apply, and/or iii) in accordance with the terms applicable for US Federal Government officers or employees acting as part of their official duties; on a worldwide, perpetual, irrevocable, royalty-free basis to BMJ Publishing Group Ltd ("BMJ") its licensees and where the relevant Journal is co-owned by BMJ to the co-owners of the Journal, to publish the Work in this journal and any other BMJ products and to exploit all rights, as set out in our licence.

The Submitting Author accepts and understands that any supply made under these terms is made by BMJ to the Submitting Author unless you are acting as an employee on behalf of your employer or a postgraduate student of an affiliated institution which is paying any applicable article publishing charge ("APC") for Open Access articles. Where the Submitting Author wishes to make the Work available on an Open Access basis (and intends to pay the relevant APC), the terms of reuse of such Open Access shall be governed by a Creative Commons licence – details of these licences and which Creative Commons licence will apply to this Work are set out in our licence referred to above.

Other than as permitted in any relevant BMJ Author's Self Archiving Policies, I confirm this Work has not been accepted for publication elsewhere, is not being considered for publication elsewhere and does not duplicate material already published. I confirm all authors consent to publication of this Work and authorise the granting of this licence.

For peer review only

Can FDG-PET/CT imaging be used to predict decline in quality of life in Interstitial Lung Disease? A prospective study of the relationship between FDG uptake and quality of life in a UK outpatient setting.

Dr Louise Jordon[1,2] *, Dr Balaji Ganeshan[3], Dr Iftikhar Nadeem[4], Luke Hoy[3], Dr Noor Mahdi[4], Prof Joanna C Porter[5,6], Prof Ashley Groves[3], Prof Thida Win[7]

[1]Department of Respiratory Medicine, Cambridge University Hospitals, Cambridge, CB2 0QQ (UK), [2]Jeffrey-Cheah Biomedical Centre, University of Cambridge, Cambridge, CB2 0AW (UK), [3]Institute of Nuclear Medicine, University College London Hospital (UCLH), London, NW1 2PG (UK), [4]Department of Respiratory Medicine Royal Papworth NHS Foundation Trust, Cambridge, CB2 0AY (UK), [5]Department of Respiratory Medicine, University College London, London, WC1E 6JF (UK), [6]ILD Centre, UCLH, London, WC1E 6JF (UK), [7]Department of Respiratory Medicine, Lister Hospital , Stevenage, SG1 4AB (UK).

*Corresponding author: Dr Louise Jordon, University of Cambridge Department of Medicine, Jeffrey Cheah Biomedical Centre, Puddicombe Way, Cambridge, CB2 0QQ. Email: louise.jordon@nhs.net, 07982713087

For peer review only

ABSTRACT

Background

^{18}F-FDG Positron Emission Tomography (PET) CT imaging has been used in many inflammatory and infectious conditions to differentiate areas of increased metabolic activity. FDG uptake differs between areas of normal lung parenchyma and interstitial lung disease (ILD).

Objectives

In this study we investigated whether FDG-PET/CT parameters were associated with change in quality of life (QoL) in patients with ILD over 4 years of follow-up.

Methods

Patients underwent PET-CT imaging at diagnosis and were followed up with annual QoL assessment using the St George's Respiratory Questionnaire until death or 4 years of follow-up. Maximum Standard Uptake Value (SUVmax) and Tissue-to-Background Ratio (TBR) were assessed against SGRQ overall and subscale scores.

Results

193 patients (94 patients in the Idiopathic Pulmonary Fibrosis; IPF subgroup and 99 patients in the non-IPF subgroup) underwent baseline FDG-PET/CT imaging and QoL assessment. Weak-to-moderate correlation was observed between baseline SUVmax and SGRQ scores in both ILD subgroups. No relationship was observed between baseline SUVmax or TBR and change in SGRQ scores over 4 years of follow-up. In the IPF subgroup, surviving patients reported a decline in QoL at 4 years post diagnosis whereas an improvement in QoL was seen in surviving patients with non-IPF ILD.

Conclusions

Weak-to-moderate positive correlation between baseline SUVmax and SGRQ scores was observed in both ILD subgroups (IPF:r_s=0.191, p=0.041, non-IPF: r_s=0.320, p=0.001). However, baseline SUVmax and TBR were not associated with change in QoL in patients with IPF and non-IPF ILD over 4 years of follow-up. At 4 years post diagnosis, surviving patients with IPF reported declining QoL whereas improvement was seen in patients with ILD who did not have IPF.

<u>Key words:</u>

Interstitial lung disease, idiopathic pulmonary fibrosis, nuclear medicine, quality of life

<u>Strengths and Limitations:</u>

Strengths:

- Large sample of patients with idiopathic pulmonary fibrosis (IPF) and non-IPF interstitial lung disease (ILD).
- 4 years of quality of life follow up data for patients with good questionnaire return rates in surviving patients

Limitations:

- Treatment and specific ILD subtype (aside from IPF) data not collected

For peer review only - http://bmjopen.bmj.com/site/about/guidelines.xhtml

INTRODUCTION

Interstitial Lung Diseases (ILDs) are a heterogenous group of pulmonary conditions characterised by varying degrees of fibrosis and inflammation of the lung parenchyma. ILDs are most frequently diagnosed by a combined clinical and radiological assessment [1]. Of these, Idiopathic Pulmonary Fibrosis (IPF) and Non-Specific Interstitial Pneumonia (NSIP) are the most frequently reported, with progressive fibrosis as the dominant histopathological lesion in IPF, and areas of inflammation and, in some cases, fibrosis occurring in NSIP [2].

Despite anti-fibrotic treatments, the prognosis for IPF is poor, with a mean survival of 2.5-5 years from diagnosis [3]. Quality of life (QoL) in IPF is affected by severe breathlessness and activity impairment [4], which demonstrates a close relationship with changes in lung function [5]. Prognosis and symptom burden in NSIP are much better, with an 86-98% 5-year survival for certain subtypes [6].

At diagnosis and during follow-up, it is difficult to predict prognosis for patients with ILD. When asked, patients have reported outcomes which they value most to be symptom burden, daily functioning and QoL [7]. Presently, decline in Forced Vital Capacity (FVC) is most frequently used to monitor disease progression [8], however baseline FVC is not predictive of change in QoL [5]. Routinely used biomarkers are lacking [9], and so tools to accurately predict QoL deterioration are needed.

Positron Emission Tomography (PET) combined with Computed Tomography (CT) offers a minimally invasive investigation to locate and quantify cellular metabolism. The most frequently used PET tracer is the glucose analogue, fluorodeoxyglucose (FDG). When labelled with the radioisotope 18-Fluorine (^{18}F), it can be used to visualise areas of regional glucose uptake and indicate metabolic activity [10-12].

Tissue metabolic activity on PET-CT is quantified through Standardised Uptake Values (SUVs) which measure the tracer's radioactivity concentration relative to the dose given and the patient's weight [13]. The SUV is most commonly reported as the maximum SUV within the region of interest (SUVmax) [14]. The tissue-to-background ratio (TBR) is a measure of variation of FDG uptake within the lung, where a value close to one indicates relatively uniform uptake [15].

In IPF lung tissue, increased glycolysis has been observed in fibroblasts [16], with increased FDG uptake seen in areas of honeycombing [17, 18]. We have previously shown that FDG uptake on PET/CT is a prognostic biomarker in IPF [19], and showed TBR to be predictive of 3 year mortality. In patients with fibrotic ILD, we have observed that TBR correlates with neoangiogenesis markers [20], and in patients with NSIP we observed that a high pulmonary background signal (SUVmin) negatively correlated with mortality [21]. Others have investigated the utility of FDG uptake in ILD subtype differentiation [22] and in determining disease activity in ILDs associated with rheumatological conditions [23].

For peer review only - http://bmjopen.bmj.com/site/about/guidelines.xhtml

The primary aim of this study was to investigate any relationship between baseline FDG-PET signal (measured by SUVmax, SUVmin and TBR) and QoL (as a self-reported questionnaire) at time of diagnosis and longitudinally over a four year follow up period in patients with ILD, to determine the utility of ^{18}F-FDG PET/CT in ILD QoL prognostication.

For peer review only

For peer review only - http://bmjopen.bmj.com/site/about/guidelines.xhtml

MATERIALS AND METHODS

Study setting and patient selection

This was a prospective, single-centre study from one UK hospital. Patients were consecutively recruited from a general respiratory clinic and enrolled for 4 years during the study period from November 2009 to December 2020. All patients with ILD who consented to having a PET-CT scan and being followed up for 4 years were eligible for inclusion in the study.

All patients underwent full clinical assessment including multidisciplinary team (MDT) review, baseline pulmonary function tests, and high-resolution CT (HRCT) evaluation. Infection and neoplasia were excluded on clinical and radiological grounds. The diagnosis of IPF was made by MDT review and was based upon HRCT findings of a "usual interstitial pneumonia" (UIP) pattern with no associated cause. If patients did not meet these diagnostic criteria, they were grouped as non-IPF ILD. Written informed consent was obtained for all participants.

Patients had an ^{18}F-FDG PET/CT scan and completed a concurrent baseline QoL questionnaire. They were subsequently followed for 48-months with QoL questionnaires at 12, 24, and 48-month intervals. Patients were excluded if they did not complete a valid QoL questionnaire at baseline, if the SUVmax and TBR could not be calculated or if a diagnosis of IPF or non-IPF ILD could not be reached. A valid questionnaire was one completed in accordance with instructions, with enough questions answered to generate a score.

Baseline demographic and pulmonary function test (PFT) data was also collected. Repeated pulmonary function tests were attempted at 12, 24 and 48 months of follow-up. Baseline ILD treatment data was available for a subset of patients. Baseline treatment was regarded as treatment prescribed within the 12 months up to and including the study commencement date.

In total, 234 patients with ILD were recruited and had ^{18}F-FDG PET/CT scans with quantification of SUVmax, SUVmin and TBR. 41 patients were excluded as they did not complete a valid baseline questionnaire, leaving 193 patients in the study.

Study objective

The primary aim of this study was to investigate the relationship between baseline ^{18}F-FDG PET/CT pulmonary uptake (SUVmax and TBR) and QoL measures, assessed using the St George's Respiratory Questionnaire (SGRQ) [24], at the time of the scan and at 12, 24 and 48 months post baseline.

Secondary objectives included looking for correlation between FDG uptake and QoL subscale scores in the total study population and in IPF and non-IPF groups, and observing QoL trajectory over the study period.

For peer review only - http://bmjopen.bmj.com/site/about/guidelines.xhtml

QoL data collection

The SGRQ is a disease-specific questionnaire designed to measure the impact on overall health, daily life, and perceived well-being in patients with respiratory disease [24, 25]. It has 50 questions divided into three sections covering: respiratory symptoms, activities that cause or are limited by breathlessness and the psycho-social impact of disease. Each question has an empirically derived weight [24], and results in a subsection and overall score out of 100, with higher scores reflecting increased severity. The SGRQ has been well validated in UK and international populations [24, 25].

Baseline SGRQ data was collected when patients attended for their ^{18}F-FDG PET/CT scan (T0), and then at 12, 24 and 48-months later (T12, T24 and T48), by postal questionnaire. QoL trends were calculated by subtracting the baseline QoL score from the score at a subsequent timepoint, thus a positive or negative score indicated a deterioration or improvement respectively.

FDG PET-CT imaging data collection

The ^{18}F-FDG PET/CT was performed as previously described [15].

Patients fasted for 6 hours. Images were acquired 1 hour after an injection of ^{18}F-FDG (200MBq) on a dedicated combined PET/64-detector CT scanner (GE Healthcare). Three sequential scans of the thorax were performed while the patient remained supine on the table with their arms above their head. First, an attenuation-correction CT (CTAC) was performed using 64 3.75 mm detectors, a pitch of 1.5, and a 5-mm collimation (140kVp and 80mA in 0.8s).

Patient position was maintained while a whole-body ^{18}F-FDG PET scan was acquired, covering an area identical to the CTAC. All images were acquired in 2-dimensional mode (8min/bed position). Transaxial emission images of 3.27mm thickness (pixel size 3.9mm) were reconstructed using ordered-subsets expectation maximization with 2 iterations and 28 subsets. The axial field of view was 148.75mm, resulting in 47 slices per bed position. Next, a deep inspiratory HRCT was performed whilst maintaining patient position, using 64 1.25 mm detectors, pitch of 0.53, and 1.25-mm collimation (120kVp and 100mAs).

Image Analysis

PET/CT images were reviewed by 2 combined radiologist/nuclear medicine physicians in consensus, one of whom had a specialist interest in cardiothoracic PET/CT. PET/CT images were loaded onto an Xeleris workstation (GE Healthcare Technology). Both attenuation and non–attenuation-corrected images were reviewed to ensure that areas of higher-density lung were not inducing attenuation-correction artifacts. The area of most intense pulmonary FDG uptake was identified and SUVmax measured. The HRCT parenchymal pattern in the region was also assessed. In addition, SUV_{min}

For peer review only - http://bmjopen.bmj.com/site/about/guidelines.xhtml

was used to determine the background lung uptake, and in turn calculate TBR.

Statistical Analysis:

Statistical analyses were performed using SPSS v26 (IBM) and Prism v8 (GraphPad). A two-sided p-value of less than 0.05 indicated a significant relationship. The relationship between FDG-PET/CT uptake and QoL was assessed using non-parametric Spearman's rank correlation.

Patient and public involvement statement:

Whilst patients were not involved directly in the design of this study, the research question and outcome measures were focused upon QoL rather than lung function or other physical parameters in line with patients' greatest priorities.

For peer review only

For peer review only - http://bmjopen.bmj.com/site/about/guidelines.xhtml

RESULTS

Total population

Baseline characteristics

In total, 193 patients met the inclusion criteria (131 males, 62 females); average age ± SD: 66.6+/-9 years). **Table 1** shows baseline characteristics

Table 1: Baseline characteristics of the study population

	Data
**Males (%)**	131 (67.8)
**Mean Age (SD)**	66.6 (9)
**Diagnosis: IPF (%)**	94 (48.7)
**NSIP (%)**	78 (40.4)
**Non-NSIP (%)**	21 (10.9)
**Mean baseline FEV1 (SD; range)**	74.9 (18.7; 17-122)
**Mean baseline FVC (SD; range)**	75.8 (17.6; 20-129)
**Mean baseline TLCO (SD; range)**	46.7 (15.5; 11-98)

The mean values for the baseline PET uptake (range in brackets) for the population were: SUVmax 3.25 (1.4-7.4), SUVmin 0.55 (0.16-1.3), and TBR 6.28 (2.4-15.3).

During follow-up of the 193 patients, 21 died by 12 months (10.9%), 42 by 24 months (21.8%) and 120 by 48 months (62.2%).

QoL assessment

QoL data was available for all 193 patients at the time of recruitment (T0), 99 patients at T12 (57.2% surviving patients), 91 patients at T24 (60.3% surviving patients) and 45 patients at T48 (61.6% surviving patients).

Table S1 (supplemental materials) demonstrates the mean QoL scores for the population who returned the SGRQ at the study time points, displayed as a percentage of the total possible score.

There was no significant difference in total SGRQ scores at T0 and T12 (t=0.44, p=0.66), T24 (t=0.36, p=0.72) or T48 (t=0.2, p=0.84).

Relationship between FDG-PET and SGRQ

Example PET-CT images with their corresponding SGRQ scores are shown in **Figure 1A-D.**

At baseline, there was weak positive correlation between SUVmax and total SGRQ score (r_s=0.229, p=0.002) and the SGRQ Symptom, Activity, and Impact subscales (r_s=0.211, 0.178 and 0.209 respectively, p<0.003 for all). There was also weak positive correlation between SUVmin and baseline total SGRQ score (r_s=0.233, p=0.001) and the SGRQ Symptom, Activity, and Impact subscales (r_s=0.161, 0.153 and 0.236

For peer review only - http://bmjopen.bmj.com/site/about/guidelines.xhtml

respectively, $p<0.05$ for all). There was no significant relationship between TBR and total (r_s=-0.015, p=0.841) and subscale SGRQ scores.

When change in QoL over time was assessed, there was no significant correlation between change in total SGRQ score and baseline SUVmax at 12 months (r_s = -0.084, p=0.403), 24 months (r_s=0.184, p=0.084) and 48 months (r_s=0.084, p=0.56). Additionally, there was no relationship between change in SGRQ subscale scores at each time point and SUVmax. There was no significant correlation between change in total SGRQ score at 12 months (r_s=-0.017, p=0.863), 24 months (r_s=0.122, p=0.253 and 48 months (r_s=0.140, p=0.332) and baseline SUVmin. We observed no significant correlation between change in total SGRQ score and baseline TBR at T12 (r_s= -0.065, p=0.519), T24 (r_s= -0.014, p=0.893) and T48 (r_s= -0.113, p=0.435), and no relationship between change in SGRQ subscale scores at each time point and TBR.

Relationship between FDG-PET, SGRQ and other parameters

Age at enrolment did not correlate with baseline SUVmax (r_s = -0.103, p=0.158) or baseline TBR (r_s= -0.033, p=0.646).

Baseline FVC and TLCO data were available for 185 and 163 patients respectively. Baseline PET parameters did not correlate with baseline PFT data, nor did baseline PET parameters correlate with change in PFT data after 12, 24 or 48 months of follow up. Further details are included in the supplemental materials (**Table S2**).

ILD treatment data was available for 127 participants at baseline (127/193; 66%). Of these, 84 participants were on no treatment, 35 were on steroid treatment, 9 were on antifibrotic treatments and 10 were on immunosuppressant treatment. 13 patients were on more than one treatment at baseline, 11 of which comprised treatments in different categories. In this subgroup of patients where treatment data was available, type of treatment (or no treatment) at baseline did not affect baseline SUVmax (H(4)=0.88, p=0.83) or SUVmin (H(4)=4.41, p=0.22). There was a significant difference in median TBR between the treatment groups (Median TBR by group: No treatment = 6.0, Steroid = 5.0, Antifibrotic = 5.3, Immunosuppressant = 4.0, H(4)=8.54, p=0.036.

There was weak negative correlation between baseline FVC and baseline TLCO and total baseline SGRQ score (FVC: r_s=-0.285, p<0.001, TLCO: r_s=-0.214,p=0.006). Change in PFT measures did not correlate with change in total SGRQ score over the 48 month follow up period.

IPF- ILD subset

Baseline demographics

There were 94 patients (84 males); average-age ± SD: 69.5+/-8.9 years) in the IPF-ILD subgroup.

The mean (range) of baseline PET metrics were: SUVmax 3.4 (1.8-6.0) and TBR 5.4 (3.1-16.6).

For peer review only - http://bmjopen.bmj.com/site/about/guidelines.xhtml

QoL assessment
QoL data was available in all 94 patients at T0. At 12 months 47 patients (50%) returned the SGRQ, 40 (42.5%) at 24 months and 22 (23.4%) at 48 months. During the follow-up period for the 94 patients, 13 died by 12 months (13.8%), 24 by 24 months (25.5%) and 74 by 48 months (78.7%).

Table S3 (supplemental materials) shows the total and subscale SGRQ scores at each time point for the IPF subgroup

When change in QoL was assessed over time, there was no significant difference in total SGRQ at T0 and T12 (t=0.74, p=0.46) or T0 and T24 (t=1.23, p=0.22) but a significant increase in total SGRQ, indicating deteriorating QoL, was seen at 48 months (t=-3.1. p=0.002).

Relationship between SGRQ scores and FDG-PET
At baseline, there was weak positive correlation between total SGRQ score and SUVmax (r_s=0.187, p=0.047; **Figure 2**). There was also weak positive correlation between the Activity SGRQ subscale and SUVmax (r_s= 0191, p=0.039). There was no significant relationship between TBR and total (r_s=0.061, p=0.524) and subscale SGRQ scores, and no significant relationship between SUVmin and total (r_s=0.107, p=0.26) and subscale SGRQ scores.

When change in QoL over time was assessed against pulmonary FDG-PET uptake, there was no significant correlation between change in total SGRQ score and baseline SUVmax at T12 (r_s=0.065, p =0.653), T24 (r_s=0.017, p=0.913) and T48 (r_s=0.084, p=0.684; Figure 1). Additionally, there was no relationship between change in SGRQ subscale scores at each time point and SUVmax, and no relationship between total or subscale SGRQ scores and SUVmin.

We also observed no significant correlation between change in total SGRQ score and baseline TBR at T12 (r_s=0.035, p=0.807), T24 (r_s= -0.122, p=0.419) and T48 (r_s=0.006, p=0.978, and no relationship between change in SGRQ subscale scores at each time point and TBR.

Non-IPF subset
Baseline demographics
There were 99 patients (45 males) in the non-IPF ILD subgroup; mean age ±SD: 64.6+/-10.1 years). The mean (range) of baseline PET uptake metrics were: SUVmax 3.1 (1.4-6.4) and TBR 6.8 (2.9-13.6).

QoL assessment
QoL data was available in all 99 patients at the time of recruitment (T0). 52 patients (52.5%) returned the QoL questionnaire at 12 months, 51 (54.3%) at 24 months and

For peer review only - http://bmjopen.bmj.com/site/about/guidelines.xhtml

23 (23.2%) at 48 months. Of the 99 patients, 8 died by 12 months (8.1%), 18 by 24 months (18.1%) and 46 by 48 months (46.5%) of follow up.

Table S4 (supplemental materials) shows the total and subscale SGRQ scores at each time point for the non-IPF subgroup

When change in QoL was assessed over time, there was no significant difference in total SGRQ at T0 and T12 (t=1.14, p=0.26) or T0 and T24 (t=1.23, p=0.22) but a significant decrease in total SGRQ, indicating improved QOL, was seen at 48 months (t=2.04. p=0.05).

In comparison with total SGRQ scores from the IPF subgroup, there was no significant difference at T0 (t=0.614, p=0.54), T12 (t=1.09, p=0.28) or T24 (t=1.43, p=0.16). Scores in the non-IPF group were significantly lower than those of the IPF group at T48 (t=-3.15, p=0.003). Comparison of overall SGRQ scores between the IPF and non-IPF subgroups is shown in **Figure 3.**

Relationship between SGRQ scores and FDG-PET

At baseline there was moderate positive correlation with total SGRQ score and SUVmax (r_s=0.320, p=0.001; **Figure 4**). There was also positive correlation between the baseline Symptoms, Activity and Impact subscale scores and baseline SUVmax (r_s= 0.287, 0.242 and 0.314, p<0.008 respectively). As baseline there was moderate positive correlation between total SGRQ and SUVmin (r_s=0.390, p=0.001). There was also positive correlation between the baseline Symptoms, Activity and Impact subscale scores and baseline SUVmin (r_s= 0.230, 0.212 and 0.366, p<0.01 respectively). There was no significant relationship between TBR and total (r_s=-0.085, p=0.379) and subscale SGRQ scores.

When change in QoL over time was assessed against pulmonary FDG PET uptake, there was no significant correlation between change in total SGRQ score and baseline SUVmax at T12 (r_s= -0.126, p=0.347), T24 (r_s=0.188, p=0.188) and T48 (r_s= -0.121, p=0.539; **Figure 3**). Additionally, there was no relationship between change in SGRQ subscale scores at each time point and baseline SUVmax, and no relationship between change in total or subscale SGRQ scores and SUVmin over time.

We also observed no significant correlation between change in total SGRQ score and baseline TBR at T12 (r_s= -0.051, p=0.702), T24 (r_s=0.075, p=0.603) and T48 (r_s=0.083, p=0.676, and no relationship between change in SGRQ subscale scores at each time point and TBR.

For peer review only - http://bmjopen.bmj.com/site/about/guidelines.xhtml

DISCUSSION

The aim of this study was to investigate the relationship between ^{18}FDG-PET/CT metrics and QoL in patients with IPF and non-IPF ILD to determine if ^{18}FDG-PET/CT could be used in ILD QoL prognostication. To our knowledge, this study is the first and largest to date investigating this relationship.

Relationship between FDG-PET/CT and QoL

Our study showed that there was a weak association between baseline SUVmax and QoL measured by the SGRQ. This may indicate that the metabolic activity of an ILD may relate to a patient's symptoms and QoL. However, in both the IPF and non-IPF subgroups, we observed no significant relationship between baseline SUVmax or TBR and change in QoL over the 48-month follow-up period. As such, these baseline PET measurements do not appear to be associated with patient-reported change in QoL for either ILD subgroup.

Work from our group has previously shown higher TBR to be predictive of survival in patients with IPF [19]. More recently we have also shown TBR to correlate with biomarkers of new vessel angiogenesis, which itself is predictive of poorer survival [26]. In 27 patients with IPF, SUVmax was shown to be predictive of FVC, TLCO and physiological decline at 12 months [18]. A larger study of 89 patients reported baseline SUVmax to be predictive of TLCO decline at 6 months, but no relationship was observed with survival outcomes [27].

However, existing literature on FDG-PET/CT as a prognostic tool in IPF is mixed. Groves *et al* [17] observed a relationship between baseline SUVmax and baseline global health and lung function in 18 patients with IPF, but these measures were not predictive of decline at 6 months. This concurs with our findings of correlation between baseline SUVmax and QoL measures in IPF patients. When evaluating the role of ^{18}F-FDG PET/CT in predicting treatment response in IPF, Bondue *et al* found SUVmax to be predictive of pirfenidone response in a mouse bleomycin model, but not in 25 patients with IPF, and nor was it predictive of disease progression during the 3 month study period in the patient population [28].

Small previous studies have shown FDG uptake distribution in NSIP, but not SUVmax to be predictive of response to treatment [29]. In patients with advanced systemic sclerosis associated ILD, ^{18}F-FDG PET/CT was unable to differentiate between areas of active inflammation (ground glass opacities on HRCT) and areas of fibrosis, but could differentiate between normal lung parenchyma and ILD changes.

To our knowledge, this is the first study evaluating the relationship between patient reported QoL and SUVmax and TBR. Whilst some studies have shown a relationship between these parameters and lung function or survival, we may not have seen a relationship between them and QoL trajectory because QoL has many more contributing factors. Furthermore, the drop out in questionnaire returns over our study period is due to patient death, and hence only patients with a milder disease trajectory were evaluated at our 48-month time point. Additionally, whilst baseline FEV1 and FVC measures of our patients were similar to those in other studies [18], our patients were enrolled from diagnosis whereas other studies enrolled subjects at later disease stages. Finally, studies evaluating PET-CT as a prognostic tool have focused on

For peer review only - http://bmjopen.bmj.com/site/about/guidelines.xhtml

different PET parameters such as FDG uptake pattern and SUVmean, and hence using these may have led to an observed relationship with QoL.

Combining this study with prior work [5, 17, 19], there is presently insufficient evidence to support using baseline FVC [5] or SUVmax/TBR to predict symptomatic decline in ILD [17], but these potentially can be used to predict survival outcomes in IPF [19].

QoL trajectory in patients with IPF and non-IPF ILD

The trajectory of patients' QoL over the follow-up period was evaluated as one of our secondary outcomes. Better QoL was observed in the non-IPF subgroup compared to the IPF group at all points from 12 to 48 months follow-up. In the IPF subgroup, QoL deterioration was observed at the 48 month time point, but not prior to this. Notably, 78% of the initial IPF cohort had died at 48 months. For the non-IPF group, there was no difference in QoL measures at 12 and 24 months compared with baseline, but a slight improvement seen at 48 months, by which point 46% of the cohort had died. Thus, patients with IPF who survived to 48 months post diagnosis noted a decline in their QoL at this stage, whereas surviving ILD patients without IPF started to improve at this stage. This enhances our knowledge of known prognostic indicators for different types of ILD. Improving QoL over the longer term in non-IPF ILD is an encouraging finding. The non-IPF ILD subgroup represent a clinically heterogenous group. The observation of improvement in QoL in surviving patients is likely due to effective treatment options for certain NSIP subtypes.

Notably, surviving patients with IPF did not note a decline in their QoL until 48 months post diagnosis. This may reflect a milder disease phenotype in the surviving patients, or signal an abrupt symptomatic decline in patients with IPF in the couple of years before they die. A large registry study of 277 IPF patients observed SGRQ-measured QoL was static for 12 months and then declined in patients a mean of three years post IPF diagnosis [30]. In particular, QoL has been shown to be very low in the two years prior to death in IPF patients [31]. Our study supports this existing work, and reinforces the need for palliative care input alongside other treatments for IPF patients at this stage of disease.

Limitations and future work

This study benefits from recruiting a large number of patients who have undergone PET-CT imaging and been followed up for 4 years. Nonetheless, there are some limitations. After baseline QoL assessment, QoL data was only available for approximately 60% of surviving patients at each subsequent time point, which could introduce nonresponse bias. Furthermore, data was not collected on specific non-IPF ILD subtypes and as such we cannot draw conclusions on specific ILDs and the effect of ^{18}F-FDG PET/CT as a prognostic marker. Finally, given the exploratory nature of this study, a formal sample size calculation was not used and as such the study could be underpowered to detect a clinically meaningful effect.

Future work could focus upon PET/CT measures and QoL in specific ILD subgroups, particularly those with an inflammatory phenotype, and could explore the role of PET-CT in treatment response prediction.

For peer review only - http://bmjopen.bmj.com/site/about/guidelines.xhtml

CONCLUSIONS

^{18}F-FDG PET/CT measure SUVmax demonstrated weak-to-moderate correlation with QoL at baseline, but baseline SUVmax and TBR were not associated with change in QoL in patients with IPF and non-IPF ILD. At present, there is insufficient evidence to support the role of ^{18}F-FDG-PET/CT in QoL prediction in ILD. Further prospective studies of ^{18}F-FDG-PET/CT involving treatment data, QoL and other imaging parameters may be beneficial to identify non-invasive disease markers.

At 4 years post IPF diagnosis, surviving patients reported declining QoL, whereas those with non-IPF ILD reported an improvement in their QoL, potentially reflecting the beneficial effect of treatment.

For peer review only

FIGURE CAPTIONS

Figure 1: Example PET-CT images from study patients. Images show the helical acquisition CT scan, attenuation-corrected F18 emission and the axially fused PET-CT (L→R). Boxed area on the scans indicates region of SUVmax. **A:** SUVmax 4.1, SGRQ 72.99, **B:** SUVmax 3.9, SGRQ 58.83, **C:** SUVmax 2.1, SGRQ 7.85, **D:** SUVmax 2.68, SGRQ 15.66.

Figure 2: Relationship between baseline SUVmax and SGRQ at (A) baseline and (B) 48 months follow up in the IPF subgroup

Figure 3: SGRQ by ILD group over 48 month follow up period.

Figure 4: Relationship between baseline SUVmax and SGRQ at (A) baseline and (B) 48 months follow up in the non-IPF subgroup

For peer review only

ACKNOWLEDGEMENTS

This work was presented at the 2022 European Respiratory Society International Congress in the ILD poster session. *Eur Respir J* 2022; 60: Suppl. 66, 2473.
https://doi.org/10.1183/13993003.congress-2022.2473

CONFLICT OF INTEREST STATEMENT

The authors report no conflicts of interest to declare.

FUNDING SOURCES

This study was undertaken at UCLH/UCL who received a proportion of funding from the Department of Health's NIHR Biomedical Research Centre's funding scheme.

STATEMENT OF ETHICS

This study was approved by the Joint UCL/UCLH Committees on the Ethics of Human Research (Committee A)

REC reference number 06/Q0505/22

DATA AVAILABILITY

Data from this study are available upon reasonable request. This comprises deidentified patient demographic and quality of life data, and calculated PET parameters. Images from which PET parameters were calculated may also be made available upon reasonable request.

AUTHOR CONTRIBUTIONS

LJ contributed to the data analysis (statistical), data visualization and drafted the original manuscript. BG contributed to data curation, data analysis (imaging and statistical), investigation, review and editing. LH contributed to data curation, investigation, visualization, review and editing. IN contributed to review and editing. NM contributed to review and editing. AG contributed to conceptualization, funding acquisition, methodology, visualization, review and editing. JP contributed to conceptualization, funding acquisition, methodology, data curation, review and editing. TW contributed to conceptualization, methodology, funding acquisition, project administration, supervision, review and editing. LJ is the submitting and corresponding author.

For peer review only

REFERENCES

[1] K.M. Antoniou, G.A. Margaritopoulos, S. Tomassetti, F. Bonella, U. Costabel, V. Poletti, Interstitial lung disease, Eur Respir Rev 23(131) (2014) 40-54.

[2] R. du Bois, T.E. King, Challenges in pulmonary fibrosis x 5: the NSIP/UIP debate, Thorax 62(11) (2007) 1008-12.

[3] H. Fujimoto, T. Kobayashi, A. Azuma, Idiopathic Pulmonary Fibrosis: Treatment and Prognosis, Clin Med Insights Circ Respir Pulm Med 9(Suppl 1) (2015) 179-185.

[4] J. De Vries, B.L. Kessels, M. Drent, Quality of life of idiopathic pulmonary fibrosis patients, Eur Respir J 17(5) (2001) 954-61.

[5] M. Kreuter, J. Swigris, D. Pittrow, S. Geier, J. Klotsche, A. Prasse, H. Wirtz, D. Koschel, S. Andreas, M. Claussen, C. Grohé, H. Wilkens, L. Hagmeyer, D. Skowasch, J.F. Meyer, J. Kirschner, S. Gläser, N. Kahn, T. Welte, C. Neurohr, M. Schwaiblmair, M. Held, T. Bahmer, T. Oqueka, M. Frankenberger, J. Behr, The clinical course of idiopathic pulmonary fibrosis and its association to quality of life over time: longitudinal data from the INSIGHTS-IPF registry, Respir Res 20(1) (2019) 59.

[6] W.D. Travis, K. Matsui, J. Moss, V.J. Ferrans, Idiopathic nonspecific interstitial pneumonia: prognostic significance of cellular and fibrosing patterns: survival comparison with usual interstitial pneumonia and desquamative interstitial pneumonia, Am J Surg Pathol 24(1) (2000) 19-33.

[7] K.I. Aronson, S.K. Danoff, A.M. Russell, C.J. Ryerson, A. Suzuki, M.S. Wijsenbeek, S. Bajwah, P. Bianchi, T.J. Corte, J.S. Lee, K.O. Lindell, T.M. Maher, F.J. Martinez, P.M. Meek, G. Raghu, G. Rouland, R. Rudell, M.M. Safford, J.S. Sheth, J.J. Swigris, Patient-centered Outcomes Research in Interstitial Lung Disease: An Official American Thoracic Society Research Statement, Am J Respir Crit Care Med 204(2) (2021) e3-e23.

[8] R.M. du Bois, D. Weycker, C. Albera, W.Z. Bradford, U. Costabel, A. Kartashov, T.E. King, L. Lancaster, P.W. Noble, S.A. Sahn, M. Thomeer, D. Valeyre, A.U. Wells, Forced vital capacity in patients with idiopathic pulmonary fibrosis: test properties and minimal clinically important difference, Am J Respir Crit Care Med 184(12) (2011) 1382-9.

[9] S. Alqalyoobi, A. Adegunsoye, A. Linderholm, C. Hrusch, C. Cutting, S.F. Ma, A. Sperling, I. Noth, M.E. Strek, J.M. Oldham, Circulating Plasma Biomarkers of Progressive Interstitial Lung Disease, Am J Respir Crit Care Med 201(2) (2020) 250-253.

[10] G.I. Bell, C.F. Burant, J. Takeda, G.W. Gould, Structure and function of mammalian facilitative sugar transporters, J Biol Chem 268(26) (1993) 19161-4.

[11] F.J. Gallagher BM , Gutterson NI , et al, Metabolic trapping as a principle of radiopharmaceutical design: some factors responsible for the biodistribution of [18 F] 2-deoxy-2-fluoro- d -glucose . Journal of Nuclear Medicine 19 (1978) 1154-1161.

For peer review only - http://bmjopen.bmj.com/site/about/guidelines.xhtml

[12] E.K. Pauwels, M.J. Ribeiro, J.H. Stoot, V.R. McCready, M. Bourguignon, B. Mazière, FDG accumulation and tumor biology, Nucl Med Biol 25(4) (1998) 317-22.

[13] P.E. Kinahan, J.W. Fletcher, Positron emission tomography-computed tomography standardized uptake values in clinical practice and assessing response to therapy, Semin Ultrasound CT MR 31(6) (2010) 496-505.

[14] M.C. Adams, T.G. Turkington, J.M. Wilson, T.Z. Wong, A systematic review of the factors affecting accuracy of SUV measurements, AJR Am J Roentgenol 195(2) (2010) 310-20.

[15] T. Win, N.J. Screaton, J.C. Porter, B. Ganeshan, T.M. Maher, F. Fraioli, R. Endozo, R.I. Shortman, L. Hurrell, B.F. Holman, K. Thielemans, A. Rashidnasab, B.F. Hutton, P.T. Lukey, A. Flynn, P.J. Ell, A.M. Groves, Pulmonary 18 F-FDG uptake helps refine current risk stratification in idiopathic pulmonary fibrosis (IPF), Eur J Nucl Med Mol Imaging 45(5) (2018) 806-815.

[16] N. Xie, Z. Tan, S. Banerjee, H. Cui, J. Ge, R.M. Liu, K. Bernard, V.J. Thannickal, G. Liu, Glycolytic Reprogramming in Myofibroblast Differentiation and Lung Fibrosis, Am J Respir Crit Care Med 192(12) (2015) 1462-74.

[17] A.M. Groves, T. Win, N.J. Screaton, M. Berovic, R. Endozo, H. Booth, I. Kayani, L.J. Menezes, J.C. Dickson, P.J. Ell, Idiopathic pulmonary fibrosis and diffuse parenchymal lung disease: implications from initial experience with 18F-FDG PET/CT, J Nucl Med 50(4) (2009) 538-45.

[18] A. Justet, A. Laurent-Bellue, G. Thabut, A. Dieudonné, M.P. Debray, R. Borie, M. Aubier, R. Lebtahi, B. Crestani, [18 F]FDG PET/CT predicts progression-free survival in patients with idiopathic pulmonary fibrosis, Respir Res 18(1) (2017) 74.

[19] T. Win, N.J. Screaton, J.C. Porter, B. Ganeshan, T.M. Maher, F. Fraioli, R. Endozo, R.I. Shortman, L. Hurrell, B.F. Holman, K. Thielemans, A. Rashidnasab, B.F. Hutton, P.T. Lukey, A. Flynn, P.J. Ell, A.M. Groves, Pulmonary 18F-FDG uptake helps refine current risk stratification in idiopathic pulmonary fibrosis (IPF), European Journal of Nuclear Medicine and Molecular Imaging 45(5) (2018) 808-818.

[20] J. Porter, B. Ganeshan, Win T, F. Fraioli, S. Khan, M. Rodrigues-Justo, R. Endozo, R. Shortman, T. Maher, A. Groves, 18F-FDG PET signal correlates with markers of neo-angiogensis in paitnets with fibrotic interstitial lung disease who underwent lung biopsy: implication for the use of PET/CT in diffuse lung diseases. , Research Square [Preprint] (2022).

[21] H. Torlot, T. Win, B. Ganeshan, N. Screaton, T. Maher, F. Fraiolo, R. Endozo, R. Shortman, A. Groves, J. Porter, S. Ratnakumar, Pulmonary 18F-FDG uptake refines current risk stratification in non-specific interstitial pneumonitis (NSIP) and predicts response to treatment., European Respiratory Journal 62 (2023).

[22] S. Nusair, R. Rubinstein, N.M. Freedman, G. Amir, N.R. Bogot, U. Izhar, R. Breuer, Positron emission tomography in interstitial lung disease, Respirology 12(6) (2007) 843-7.

For peer review only - http://bmjopen.bmj.com/site/about/guidelines.xhtml

[23] D.M. Peelen, B.G.J.C. Zwezerijnen, E.J. Nossent, L.J. Meijboom, O.S. Hoekstra, C.J. Van der Laken, A.E. Voskuyl, The quantitative assessment of interstitial lung disease with positron emission tomography scanning in systemic sclerosis patients, Rheumatology (Oxford) 59(6) (2020) 1407-1415.

[24] P.W. Jones, F.H. Quirk, C.M. Baveystock, The St George's Respiratory Questionnaire, Respir Med 85 Suppl B (1991) 25-31; discussion 33-7.

[25] M. Ferrer, C. Villasante, J. Alonso, V. Sobradillo, R. Gabriel, G. Vilagut, J.F. Masa, J.L. Viejo, C.A. Jiménez-Ruiz, M. Miravitlles, Interpretation of quality of life scores from the St George's Respiratory Questionnaire, Eur Respir J 19(3) (2002) 405-13.

[26] J. Porter, B. Ganeshan, T. Win, F. Fraioli, S. Khan, M. Rodriguez-Justo, R. Endozo, R. Shortman, T. Maher, A. Groves, [18 F]FDG PET signal correlates with markers of neo-angiogenesis in patients with fibrotic interstitial lung disease who underwent lung biopsy: Implication for the use of PET/CT in diffuse lung diseases, Preprint: Research Square (2022).

[27] Hee-Young Yoon, Suk Hyun Lee, Sejin Ha , Jin-Sook Ryu , J.W. Song, The Value of 18 F-FDG PET/CT in Evaluating Disease Severity and Prognosis in Idiopathic Pulmonary Fibrosis Patients, 2021, p. 257.

[28] B. Bondue, A. Castiaux, G. Van Simaeys, C. Mathey, F. Sherer, D. Egrise, S. Lacroix, F. Huaux, G. Doumont, S. Goldman, Absence of early metabolic response assessed by 18F-FDG PET/CT after initiation of antifibrotic drugs in IPF patients, Respir Res 20(1) (2019) 10.

[29] V. Jacquelin, A. Mekinian, P.Y. Brillet, H. Nunes, O. Fain, D. Valeyre, M. Soussan, FDG-PET/CT in the prediction of pulmonary function improvement in nonspecific interstitial pneumonia. A Pilot Study, Eur J Radiol 85(12) (2016) 2200-2205.

[30] W.A. Wuyts, C. Dahlqvist, H. Slabbynck, M. Schlesser, N. Gusbin, C. Compere, S. Maddens, S. Rizzo, K.U. Kirchgaessler, K. Bartley, B. Bondue, Quality of Life and Healthcare Resource Use in a Real-world Patient Population with Idiopathic Pulmonary Fibrosis: The PROOF Registry, Pulm Ther 8(2) (2022) 181-194.

[31] K. Rajala, J.T. Lehto, E. Sutinen, H. Kautiainen, M. Myllärniemi, T. Saarto, Marked deterioration in the quality of life of patients with idiopathic pulmonary fibrosis during the last two years of life, BMC Pulm Med 18(1) (2018) 172.

For peer review only

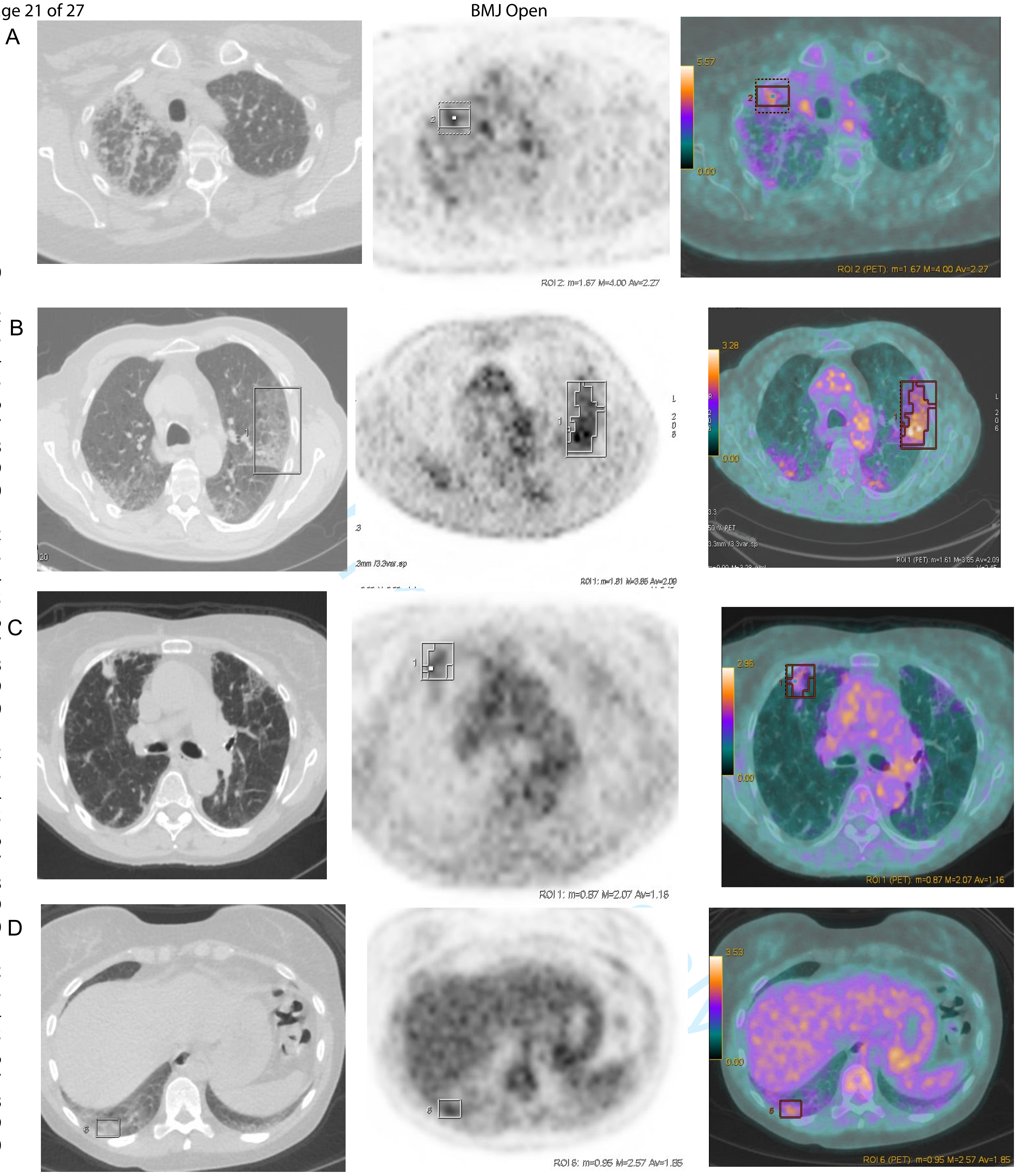

Figure 1: Example PET-CT images from study patients. Images show the helical acquisition CT scan, attenuation-corrected F18 emission and the axially fused PET-CT (L→R). Boxed area on the scans indicates region of SUVmax. **A:** SUVmax 4.1, SGRQ 72.99, **B:** SUVmax 3.9, SGRQ 3.9, **C:** SUVmax 2.1, SGRQ 7.85, **D:** SUVmax 2.68, SGRQ 15.66.

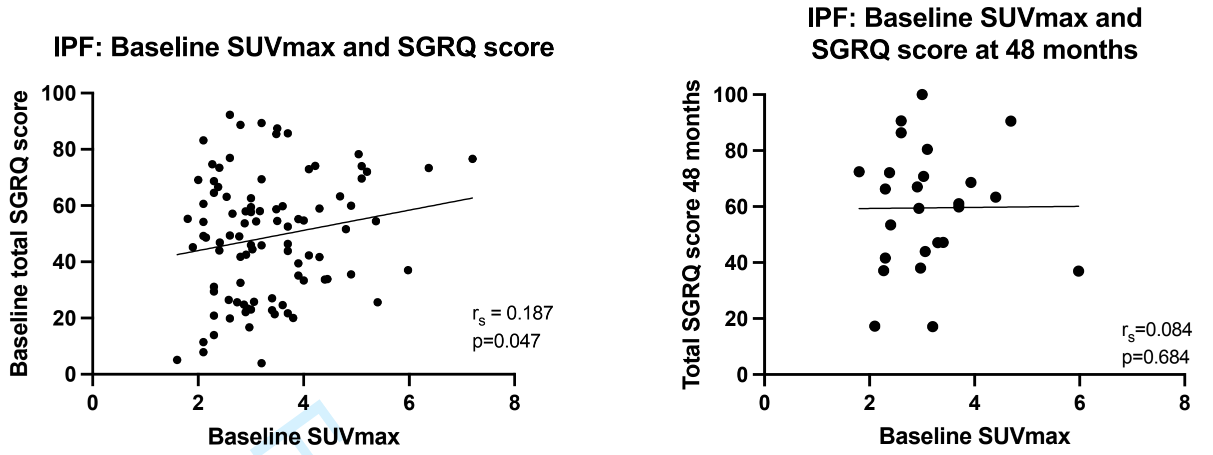

Figure 2: Relationship between baseline SUVmax and SGRQ at (A) baseline and (B) 48 months follow up in the IPF subgroup

For peer review only

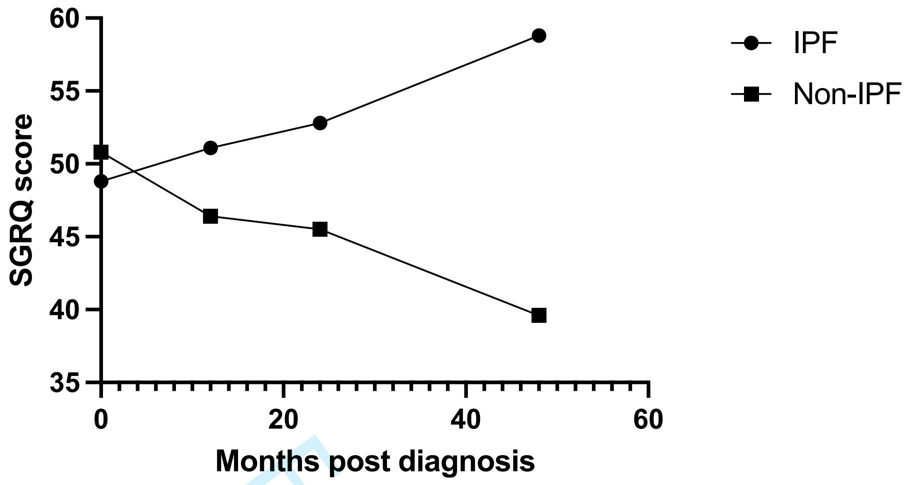

Figure 3: SGRQ by ILD group over 48 month follow up period.

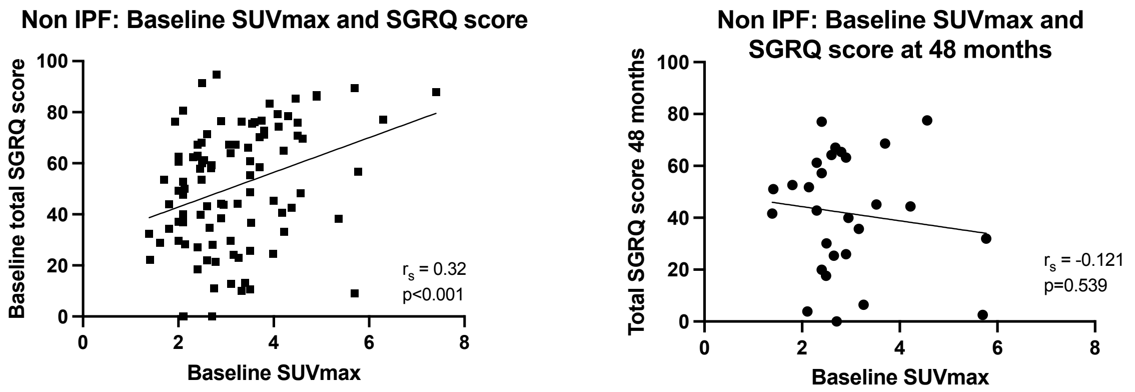

Figure 4: Relationship between baseline SUVmax and SGRQ at (A) baseline and (B) 48 months follow up in the non-IPF subgroup

For peer review only

Supplemental Materials

Table S1: Mean SGRQ scores for the total study population at each time point

	Total	Symptoms	Activity	Impact
**T0**	49.8	54.7	64.1	39.9
**T12**	48.6	51.0	64.7	36.4
**T24**	48.7	50.8	64.2	38.3
**T48**	48.9	52.2	66.2	39.4

Table S2: Correlation between change in PFTs from baseline and baseline FDG-PET parameters

**Correlation between change in FVC from baseline and SUVmax (number of participants)**		
	**Correlation Coefficient (r_s)**	**P value**
**Baseline** (185)	-0.176	0.017
**12 months** (119)	0.010	0.912
**24 months** (73)	0.144	0.225
**48 months** (15)	0.220	0.427
**Correlation between change in TLCO from baseline and SUVmax (number of participants)**		
	**Correlation Coefficient (r_s)**	**P value**
**Baseline** (163)	-0.158	0.043
**12 months** (90)	0.072	0.503
**24 months** (52)	0.030	0.831
**48 months** (10)	0.185	0.604
**Correlation between change in FVC from baseline and TBR (number of participants)**		
	**Correlation Coefficient (r_s)**	**P value**
**Baseline** (185)	-0.003	0.967
**12 months** (119)	-0.101	0.273
**24 months** (73)	0.027	0.818
**48 months** (15)	-0.143	0.611
**Correlation between change in TLCO from baseline and TBR (number of participants)**		
	**Correlation Coefficient (r_s)**	**P value**
**Baseline** (163)	-0.036	0.648
**12 months** (90)	-0.052	0.625
**24 months** (52)	-0.046	0.748
**48 months** (10)	-0.098	0.789

Table S3: Total and subscale SGRQ scores at each time point for the IPF subgroup

	Total	Symptoms	Activity	Impact
**T0**	48.8	57.1	63.1	37.8
**T12**	51.1	58.5	66.1	39.3
**T24**	52.8	56.6	67.7	42.8
**T48**	58.8	60.8	72.7	48.7

For peer review only

Table S4: Total and subscale SGRQ scores at each time point for the non-IPF subgroup

	Total	Symptoms	Activity	Impact
**T0**	50.8	52.3	65.1	41.9
**T12**	46.4	44.6	63.3	33.8
**T24**	45.5	46.5	61.6	35.3
**T48**	39.6	43.4	59.1	30.1

For peer review only

STROBE Statement—Checklist of items that should be included in reports of ***cohort studies***

	Item No	Recommendation	Page No
**Title and abstract**	1	(*a*) Indicate the study's design with a commonly used term in the title or the abstract<br>(*b*) Provide in the abstract an informative and balanced summary of what was done and what was found	1
**Introduction**			
Background/rationale	2	Explain the scientific background and rationale for the investigation being reported	2
Objectives	3	State specific objectives, including any prespecified hypotheses	2-4
**Methods**			
Study design	4	Present key elements of study design early in the paper	4-5
Setting	5	Describe the setting, locations, and relevant dates, including periods of recruitment, exposure, follow-up, and data collection	4-5
Participants	6	(*a*) Give the eligibility criteria, and the sources and methods of selection of participants. Describe methods of follow-up<br>(*b*) For matched studies, give matching criteria and number of exposed and unexposed	4
Variables	7	Clearly define all outcomes, exposures, predictors, potential confounders, and effect modifiers. Give diagnostic criteria, if applicable	4,5
Data sources/ measurement	8*	For each variable of interest, give sources of data and details of methods of assessment (measurement). Describe comparability of assessment methods if there is more than one group	4,5
Bias	9	Describe any efforts to address potential sources of bias	5-6
Study size	10	Explain how the study size was arrived at	
Quantitative variables	11	Explain how quantitative variables were handled in the analyses. If applicable, describe which groupings were chosen and why	5-6
Statistical methods	12	(*a*) Describe all statistical methods, including those used to control for confounding<br>(*b*) Describe any methods used to examine subgroups and interactions<br>(*c*) Explain how missing data were addressed<br>(*d*) If applicable, explain how loss to follow-up was addressed<br>(*e*) Describe any sensitivity analyses	5-6
**Results**			
Participants	13*	(a) Report numbers of individuals at each stage of study—eg numbers potentially eligible, examined for eligibility, confirmed eligible, included in the study, completing follow-up, and analysed<br>(b) Give reasons for non-participation at each stage<br>(c) Consider use of a flow diagram	7
Descriptive data	14*	(a) Give characteristics of study participants (eg demographic, clinical, social) and information on exposures and potential confounders<br>(b) Indicate number of participants with missing data for each variable of interest<br>(c) Summarise follow-up time (eg, average and total amount)	7-9
Outcome data	15*	Report numbers of outcome events or summary measures over time	7-9

For peer review only

For peer review only - http://bmjopen.bmj.com/site/about/guidelines.xhtml

Main results	16	(*a*) Give unadjusted estimates and, if applicable, confounder-adjusted estimates and their precision (eg, 95% confidence interval). Make clear which confounders were adjusted for and why they were included<br>(*b*) Report category boundaries when continuous variables were categorized<br>(*c*) If relevant, consider translating estimates of relative risk into absolute risk for a meaningful time period	7-9
Other analyses	17	Report other analyses done—eg analyses of subgroups and interactions, and sensitivity analyses	7-10
**Discussion**			
Key results	18	Summarise key results with reference to study objectives	11
Limitations	19	Discuss limitations of the study, taking into account sources of potential bias or imprecision. Discuss both direction and magnitude of any potential bias	12
Interpretation	20	Give a cautious overall interpretation of results considering objectives, limitations, multiplicity of analyses, results from similar studies, and other relevant evidence	11-12
Generalisability	21	Discuss the generalisability (external validity) of the study results	11-13
**Other information**			
Funding	22	Give the source of funding and the role of the funders for the present study and, if applicable, for the original study on which the present article is based	15

*Give information separately for exposed and unexposed groups.

Note: An Explanation and Elaboration article discusses each checklist item and gives methodological background and published examples of transparent reporting. The STROBE checklist is best used in conjunction with this article (freely available on the Web sites of PLoS Medicine at http://www.plosmedicine.org/, Annals of Internal Medicine at http://www.annals.org/, and Epidemiology at http://www.epidem.com/). Information on the STROBE Initiative is available at http://www.strobe-statement.org.

For peer review only
